# Unveiling the Mechanism of Compound Ku-Shen Injection in Liver Cancer Treatment through an Ingredient–Target Network Analysis

**DOI:** 10.3390/genes15101278

**Published:** 2024-09-29

**Authors:** Wenkui Zou, Jiazhen Liu, Zexing Wei, Chunhua Peng, Ying Zhao, Yue Ding, Jifan Shi, Juan Zhao

**Affiliations:** 1 School of Pharmacy, Shanghai University of Traditional Chinese Medicine, Shanghai 201203, China; 2 School of Traditional Chinese Medicine, Shanghai University of Traditional Chinese Medicine, Shanghai 201203, China; 3 Research Institute of Intelligent Complex Systems, Fudan University, Shanghai 200433, China; 4 Shanghai Artificial Intelligence Laboratory, Shanghai 200232, China

**Keywords:** compound matrine injection, network pharmacology, liver cancer, molecular docking

## Abstract

Background: Compound Ku-Shen Injection (CKI) is a traditional Chinese medicine preparation derived from Ku-Shen and Bai-Tu-Ling, commonly used in the adjunctive treatment of advanced cancers, including liver cancer. However, the underlying mechanisms of CKI’s effectiveness in cancer treatment are not well defined. Methods: This study employs network pharmacology to investigate the traditional Chinese medicine (TCM) compatibility theory underlying CKI’s action in treating liver cancer, with findings substantiated by molecular docking and in vitro experiments. Sixteen active components were identified from CKI, along with 193 potential targets for treating liver cancer. Key therapeutic target proteins, including EGFR and ESR1, were also identified. KEGG enrichment results showed that the neuroactive ligand–receptor interaction, cAMP signaling pathway, and serotonergic synapses make up the key pathway of CKI in the treatment of liver cancer. Molecular docking results confirmed that the key active ingredients effectively bind to the core targets. CCK-8 cytotoxic experiment results show that the CKI key components of oxymatrine and matrine can inhibit the growth of HepG2 liver cancer cell proliferation. A Western blot analysis revealed that oxymatrine suppresses the expression of EGFR, contributing to its therapeutic efficacy against liver cancer. Conclusion: our study elucidated the therapeutic mechanism of CKI in treating liver cancer and unveiled the underlying principles of its TCM compatibility through its mode of action.

## 1. Introduction

Liver cancer is one of the most common and deadly malignant tumors in modern society, with its incidence increasing annually [1]. Liver cancer is marked by an insidious onset, rapid progression, high malignancy, and significant mortality. Currently, the primary treatments for liver cancer include surgery, chemotherapy, biological therapy, and radiotherapy [2]. Although these treatments can be effective for liver cancer patients, they also carry significant side effects. As research in traditional Chinese medicine (TCM) advances [3], using TCM alone or in combination with anticancer drugs to treat liver cancer has gained increasing attention [4]. This approach is particularly noted for reducing drug toxicity, enhancing treatment effectiveness, improving patient quality of life, and extending survival [5].

Compound Ku-Shen Injection (CKI) is a TCM preparation extracted from Ku-Shen and Bai-Tu-Ling, known for its functions of clearing heat and dampness, cooling blood, detoxifying, dispersing condensation, and alleviating pain [6]. CKI has been used clinically for over 15 years in China to treat cancer-related pain and bleeding [7]. It is widely employed in the adjuvant treatment of advanced cancers, including recurrent liver cancer, gastric cancer, and non-small cell lung cancer [8], and is also used for analgesia and hemostasis. CKI is recognized as one of the Chinese patent medicines with notable clinical efficacy [9].

As a complex traditional Chinese medicine compound, CKI presents challenges in traditional pharmacological research, making its mechanism of action difficult to fully elucidate [10]. Exploring the molecular mechanisms and internal compatibility of CKI in treating liver cancer is crucial for scientifically interpreting TCM theories and broadening CKI’s clinical application [11]. Network pharmacology builds network models by connecting various nodes, translating complex biological system interactions into intuitive network maps [12]. This approach facilitates the identification and understanding of biological systems by analyzing the composition and characteristics of these maps, offering inherent advantages in uncovering the mechanisms of action of traditional Chinese medicine compounds [13]. Therefore, this study employed network pharmacology to identify the active components of CKI, and analyze its targets, and related signaling pathways in treating liver cancer [14]. We validated the accuracy of our component and target screening through molecular docking and in vitro experiments, laying the groundwork for further research.

## 2. Materials and Methods

### 2.1. Identification of the Active Components and Targets of CKI 

We used the Chinese Medicine System Pharmacology database and analysis platform (https://tcmsp-e.com/tcmsp.php, accessed on 1 January 2024) [15] to screen two Chinese herbs (Ku-Shen and Bai-Tu-Ling) in the formula of CKI and searched the active ingredients in the formula through the literature. The active ingredients of CKI were evaluated based on two main indicators, oral bioavailability (OB) and drug similarity (DL), with screening thresholds set at OB ≥ 30% and DL ≥ 0.18, respectively. We entered the active components in CKI into the PubChem database (https://pubchem.ncbi.nlm.nih.gov/, accessed on 1 January 2024) to obtain their SMILES structures [16]. The obtained CKI compound SMILES structures were uploaded to the pharmacodynamic Prediction target database (Swiss Target Prediction, http://www.swisstargetprediction.ch/, accessed on 1 January 2024) [17], the species was selected as “Homo sapiens”, and the target-prediction results were ranked from the highest probability to the lowest. To ensure the accuracy of the screening results, we selected the targets with a probability greater than 0.05, and excluded the target proteins that lacked the corresponding gene name.

### 2.2. Target Selection for Liver Cancer

Target selection for liver cancer was conducted using the Online Mendelian Inheritance in Man (https://www.OMIM.org/, accessed on 1 January 2024) [18] and Gene Cards database (https://www.genecards.org/, accessed on 1 January 2024) [19], with “liver cancer” as the keyword. Targets from these databases were normalized and standardized. Target proteins lacking the corresponding gene names were excluded. After eliminating duplications, the specific targets associated with liver cancer were identified.

### 2.3. Identifying the Potential Targets of CKI for Liver Cancer Treatment

The potential therapeutic targets of CKI and those associated with liver cancer were identified by using an online Venn diagram tool to find common targets. The Venn diagram visually displays these intersections, facilitating the exploration of CKI’s mechanisms of action in treating liver cancer.

### 2.4. Construction of Ingredient–Target Network

We imported the shared targets of drugs and diseases, along with the core components of drugs, into Cytoscape 3.9.1 software to construct the “component–target” network. Then, we conducted a topological parameter analysis to identify the core genes.

### 2.5. Construction of Protein–Protein Interaction (PPI) Network

We imported the potential intersecting targets of CKI for liver cancer treatment into the STRING database (https://STRING-db.org/, accessed on 1 January 2024) [20] as gene symbols, setting the species restriction to “Homo sapiens”. The STRING database was used to analyze the interaction among the targets of CKI in treating liver cancer, and the proteins with a confidence score ≥ 0.90 were selected, and any free nodes were removed to construct the protein–protein interaction (PPI) network.

### 2.6. Gene Ontology (GO) and Kyoto Encyclopedia of Genes and Genomes (KEGG) Pathway Enrichment Analyses

The identified potential therapeutic target genes were subjected to GO functional annotation and a KEGG pathway enrichment analysis to elucidate the potential mechanism of CKI in treating liver cancer. A GO enrichment analysis annotates genes across three dimensions, the biological process (BP), cellular component (CC), and molecular function (MF). The KEGG pathway enrichment analysis was performed specifically for the “human” species, requiring each pathway to include at least three distinct genes. The results were visualized using bar and bubble plots.

### 2.7. Molecular Docking

The targets from the PPI network and the top three core components from the “component–target” were selected as receptor proteins and ligand small molecules. The crystal structure of the receptor protein was retrieved from the PDB database (https://www.rcsb.org/structure/, accessed on 1 January 2024) and their PDB format files were downloaded. Ligand molecules in MOL2 format were downloaded from the PubChem database (https://pubchem.ncbi.nlm.nih.gov/, accessed on 1 January 2024) [16]. Prior to binding, we conducted operations including adding hydrogen to the macromolecule, calculating charge numbers, and determining the rigid properties of atoms to ensure a clean protein structure. Molecular docking of the ligands and receptors was performed using Auto Dock Tools 1.5.7, and the results were visualized to assess the interactions [21]. We obtained the Protein Data Bank (PDB) files (5I6Z, 3DT3) for the core targets EGFR and ESR1 from PDB. Molecular docking experiments were performed using these PDB files. The co-crystallized ligands involved were CID_114850 and CID_91466. The resolutions of the PDB structures 5I6Z and 3DT3 were 2.4 Angstroms (Å) and 2.0 Å, respectively. Additionally, we also validated our method through re-docking. We used 2.0 Å as the limit for Root Mean Square Deviation (RMSD) in the re-docking, which is typically used to indicate a satisfactory alignment of a molecular model with the reference structure [22]. When conducting molecular docking, we also collected the relevant parameters of the virtual box to validate the results (Table 1).

### 2.8. In Vitro Experiments

#### 2.8.1. Cell Culture

The human hepatoma cell line HepG2 was purchased from the Stem Cell Bank of the Chinese Academy of Sciences. The cells were cultured in an MEM medium containing 10% fetal bovine serum, 100 U/mL penicillin, and 100 U/mL streptomycin at 37 °C and 5% CO_2_.

#### 2.8.2. Reagents and Instruments

The MEM culture medium (gibco, Shanghai, China), fetal bovine serum (Sigma-Aldrich, F0193, Darmstadt, Germany), DMSO (MedChemExpress, Darmstadt, Germany), cell lysate, CCK-8, BCA protein quantification kit (Jiangsu Biyantian Biological Technology Institute, Changzhou, China), PVDF membrane, GAPDH antibody, EGFR antibody, Phospho-EGFR antibody (Abcam, Shanghai, China), matrine, oxymatrine (Shanghai Yuanye Biotechnology Co., Ltd., Shanghai, China), CO_2_ constant-temperature incubator, ultra-clean table, high-speed centrifuge, and microplate reader (TECAN SPARK company, Männedorf, Switzerland) were among the reagents and instruments used.

#### 2.8.3. Cell Proliferation Detection

HepG2 cells in the logarithmic growth phase were digested with trypsin and counted, and seeded in 96-well plates at a concentration of 8 × 10^3^/well. After 24 h of culture, the medium was discarded and replaced with a medium containing different concentrations of oxymatrine and matrine. After continued culture for 48 h, the drug-containing medium was discarded, and 100 µL of a medium containing a 10% CCK-8 reagent was added to each well. After continued incubation in the incubator for 1 h, the OD value at 450 nm was detected by the microplate reader, and the cell survival rate (%) = (A sample-A blank)/(A control-A blank) × 100%.

#### 2.8.4. Western Blotting

HepG2 cells in the logarithmic growth phase were seeded in 6-well plates at a cell density of 1 × 10^5^ cells/well. After the cells adhered to the wall for stable growth for 24 h overnight, the culture medium was discarded; 2 μM oxymatrine and 2 μM gefitinib were added to the wells. The lysate was centrifuged at 4 °C to extract the supernatant, and the total protein was quantified and characterized. Then, the sample was transferred to the PVDF membrane, blocked with BSA, incubated with a primary antibody overnight, incubated with a secondary antibody, washed membrane-wise, and developed. GAPDH was used as the internal reference protein, and Image J (ImageJ bundled with 64-bit Java 8) was used to analyze the gray value of the band, and the relative expression level was recorded.

#### 2.8.5. Statistical Methods

The data were expressed by $\bar{x}\pm s$ and analyzed by SPSS26.0 statistical software. The comparison between the two groups was performed by a *t* test, and *p* < 0.05 was considered statistically significant.

## 3. Results

### 3.1. Identification of Active Ingredients and Targets of CKI

Twenty-one active ingredients of CKI were initially identified through the literature and database searches [23]. However, a query in the PubChem database revealed only 16 of these ingredients. Consequently, these 16 active ingredients were used for subsequent network pharmacology research (Table 2). The targets of CKI were predicted based on the structure of these 16 active components, with therapeutic targets of CKI being identified in the Swiss Target Prediction database. After removing duplicates, a total of 220 targets were obtained.

### 3.2. Identification of Target for Liver Cancer

We searched the OMIM database and Gene Cards database using “liver cancer” as the keyword. The target data from these databases were compiled and normalized. After excluding target proteins without corresponding gene names, a total of 18,174 liver cancer targets were identified.

### 3.3. Potential Targets of CKI for Liver Cancer Treatment

**Figure 1:**
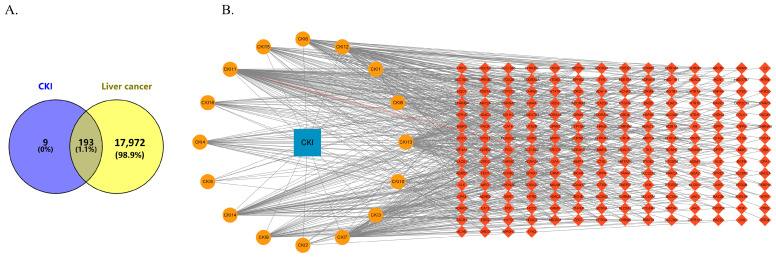
(**A**) The Venn diagram of overlapping target genes of CKI and liver cancer. (**B**) The ingredient–target network of CKI, where the blue rectangle is CKI, the circular nodes represent the active components of CKI, and the prism nodes denote the potential targets of CKI.

After a thorough analysis, we successfully identified 220 target proteins related to the drug’s mechanisms of action and 18,174 target proteins associated with liver cancer. Using online software mapping tool platform Venny 2.1 (https://bioinfogp.cnb.csic.es/tools/venny/index.html, accessed on 1 January 2024), we inputted the target data for both CKI and liver cancer. This mapping resulted in the identification of 193 potential target proteins for CKI in liver cancer treatment (Figure 1A).

### 3.4. Ingredient–Target Network Analyses

Cytoscape 3.9.1 software was utilized to create and analyze the component–target network diagram for CKI in the treatment of liver cancer, revealing the relationship between various active components and core targets. As illustrated in Figure 1B, the circular nodes represent the active components of CKI while the prism nodes denote the potential targets of CKI for liver cancer treatment. Within the network map, oxymatrine, isomatrine, and matrine were identified as key components in the treatment of liver cancer.

### 3.5. Protein–Protein Interaction (PPI) Network Analyses

**Figure 2:**
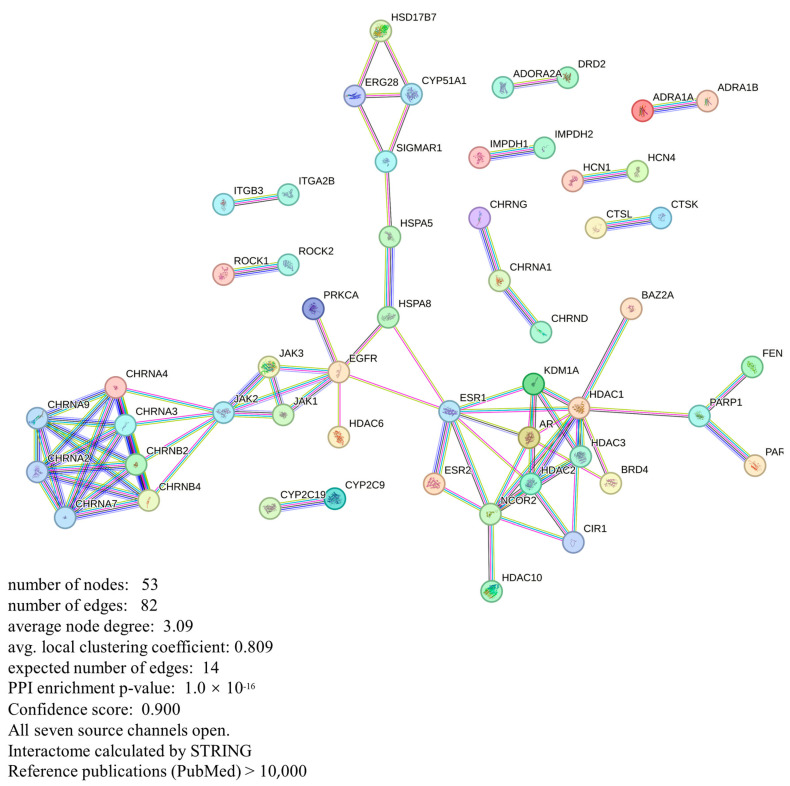
Protein–protein interaction network of CKI.

We imported the 193 intersecting targets identified through screening into the STRING database (https://STRING-db.org/, accessed on 1 January 2024) as gene symbols, specifying “Homo sapiens” as the restricted species. In the STRING analysis of CKI, we selected interactions with a confidence score of 0.90 and eliminated any free nodes from the network. This process yielded a protein–protein interaction (PPI) network consisting of 192 nodes and 157 connecting lines. The quantitative evaluation of the composition of the individual interactions calculated by STRING is available in Supplementary Material S1 (Table S1). We observed that numerous proteins, although assigned high confidence scores, lack experimental validation and corresponding experimental scores based on node information from the STRING database. To enhance the accuracy of our data, we excluded proteins and interactions with experimentally determined scores below 0.4. Consequently, this filtration resulted in a protein–protein interaction (PPI) network comprising 53 nodes and 82 connections, as depicted in Figure 2. Cytoscape 3.9.1 software was used to analyze the topology of the network by using the “CytoNCA” plug-in. According to the results of the topological parameter analysis, the target proteins in the PPI network were ranked by the degree value (Table 3). The results showed that Histone Deacetylase 1 (HDAC1), Estrogen Receptor 1 (ESR1), and epidermal growth factor receptor (EGFR) ranked high overall. Although HDAC1 ranks first in terms of the degree value in the protein network results, its betweenness centrality (BC) value is relatively low. As a result, ESR1 and EGFR were identified as potential core targets of CKI for treating liver cancer and were selected for a subsequent experimental analysis.

### 3.6. GO and KEGG Pathway Enrichment Analyses

**Figure 3:**
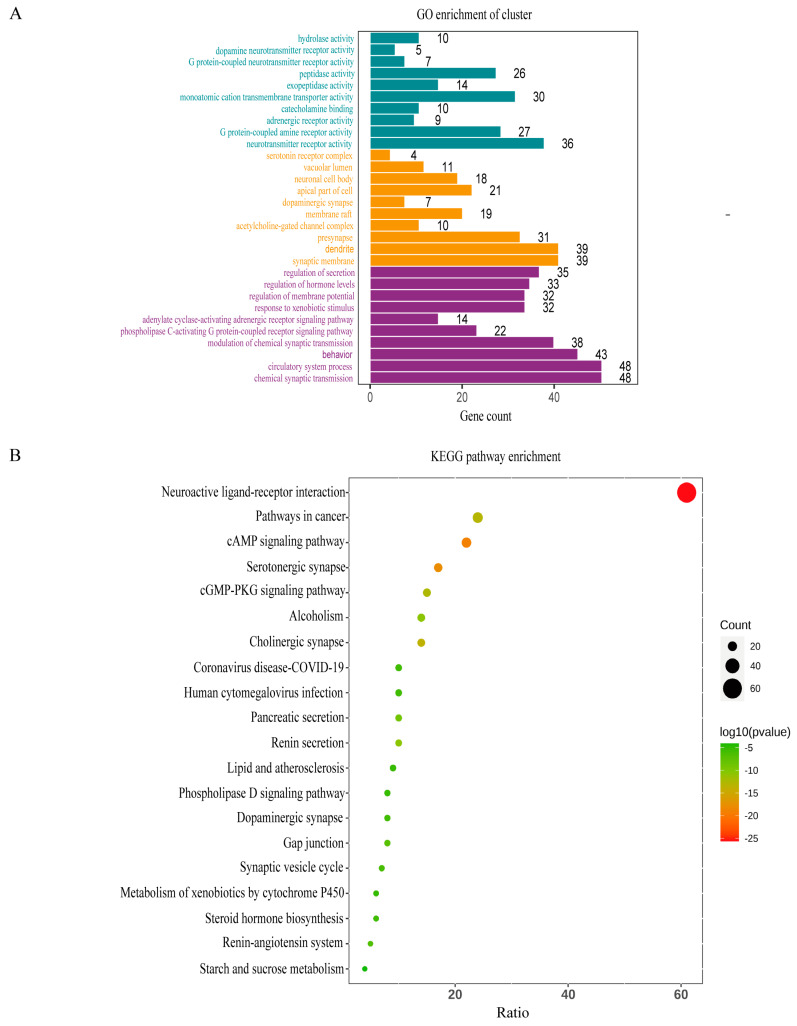
(**A**) Bar graph of GO enrichment results, organized vertically from top to bottom: MF, CC, BP. (**B**) KEGG pathway enrichment results.

GO enrichment results revealed that CKI treatment for liver cancer primarily affects biological processes such as neurotransmitter receptor activity and G protein-coupled amine receptor activity and components like the synaptic membrane, dendrite, and chemical synaptic transmission and circulatory system processes (Figure 3A). According to the KEGG enrichment results (*p* < 0.01), the top 20 most significant pathways (Figure 3B) include the neuroactive ligand–receptor interaction, cAMP signaling pathway, and serotonergic synapse, among others.

### 3.7. Molecular Docking Results

EGFR, ESR1, and the top three active ingredients from the component–target network and PPI network—matrine, oxymatrine, and isomatrine—each ranked by the median value, were selected for molecular docking using Auto-dock software (AutoDockTools-1.5.7).

Based on the re-docking result, when compared with the crystal structures, the re-docking RMSD values for the three compounds were 1.61 Å and 1.41 Å. As all RMSD values are below 2.0 Å, this demonstrates that the molecular docking method and parameter settings are reliable. Additional details and re-docking documentation, including information on each protein, protein–ligand interactions, and RMSD results, are available in Supplementary Material (S2).

**Figure 4:**
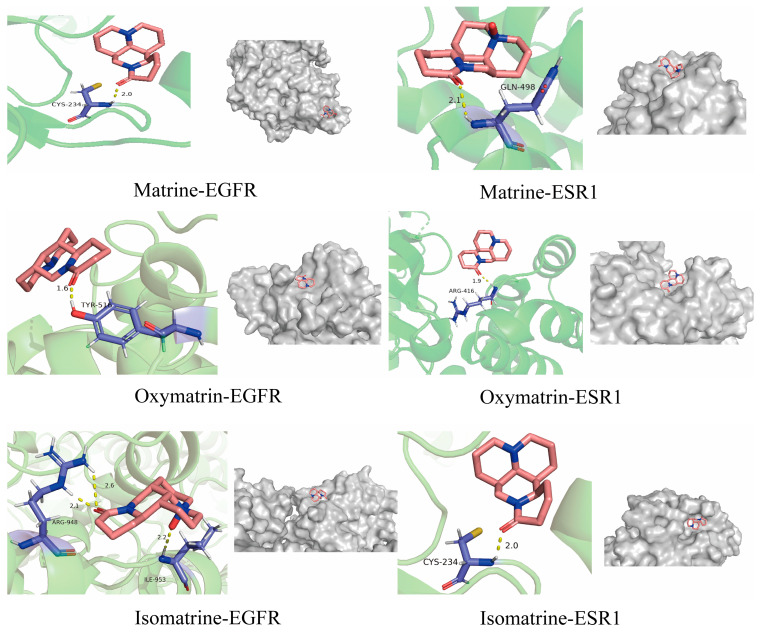
Molecular docking profiles of active ingredients and core targets.

The docking results are presented in Table 4, with visualizations created using PyMOL (Figure 4). These results demonstrate that the binding energies of the top three active ingredients to the core protein targets were all below −5 kcal/mol, indicating strong affinity [21,24]. Further documentation and details of the molecular docking process are available in Supplementary Material (S3).

### 3.8. Effect of Oxymatrine and Matrine on Cell Proliferation

**Figure 5:**
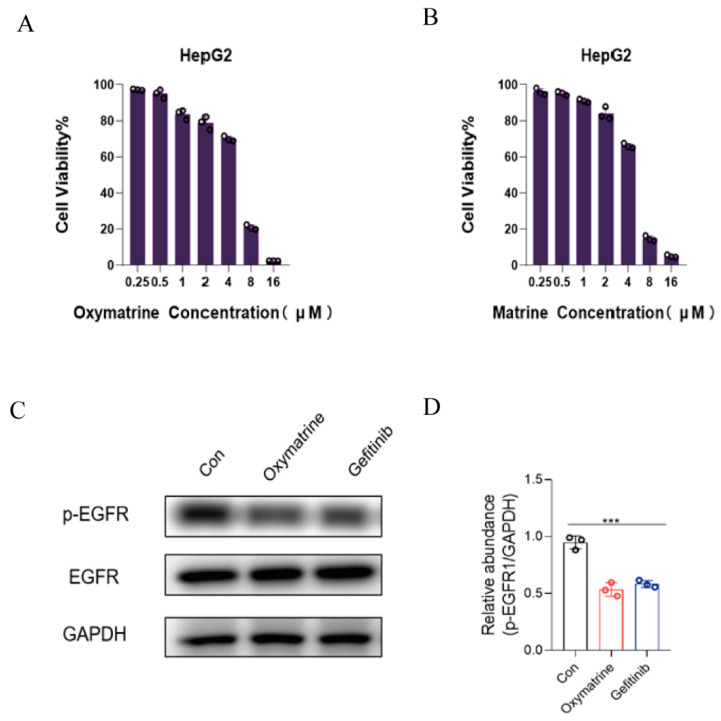
(**A**,**B**) Effects of matrine and oxymatrine on the proliferation of HepG2 cells. (**C**,**D**) The effect of oxymatrine on EGFR expression in HepG2 cells. All the results are shown as mean ± SD (n = 3), *** *p* < 0.001.

After 48 h of treatment with oxymatrine and matrine, the CCK-8 assay indicated that both compounds significantly reduced the growth and proliferation of HepG2 cells. The 50% inhibitory concentration (IC50) of oxymatrine on HepG2 cells was 5.794 ± 0.763 Μm, and the 50% inhibitory concentration (IC50) of matrine on HepG2 cells was 4.512 ± 0.654 μM (Figure 5A,B).

### 3.9. Effect of Oxymatrine on the Expression of EGFR

Based on the molecular docking results, oxymatrine demonstrated the lowest binding energy with the EGFR protein target. Consequently, we employed a Western blot analysis to assess oxymatrine’s impact on the expression of phosphorylated EGFR (p-EGFR). Western blot results showed that oxymatrine could reduce the expression of p-EGFR in HepG2 cells and achieve a therapeutic effect on liver cancer (Figure 5C,D).

## 4. Discussion

CKI has a longstanding history and has demonstrated effectiveness in the clinical treatment of various types of cancer, exhibiting definite therapeutic effects without significant adverse reactions [25]. In our study, we discovered that most of the drug’s core components are derived from the traditional Chinese medicine Ku-Shen [26]. These components from Ku-Shen exhibited a potent cytotoxic effect on liver cancer cells. In contrast, the components from Bai-Tu-Ling did not demonstrate a direct cytotoxic effect on liver cancer cells. Shen et al. [8] utilized a transcriptome analysis to study the effects and interactions of two plant extracts, sophora and clay three, in conjunction with CKI. By comparing the components and effects of these two individual traditional Chinese medicines and compound drugs [27], we elucidated the drug compatibility at the deep molecular level. It was discovered that Ku-Shen played a pivotal role in CKI; the cytotoxicity and cellular effect of CKI were primarily derived from Ku-Shen. Meanwhile, Bing-Tu-Ling mainly serves to enhance Ku-Shen’s potential anticancer effects and activate the immune system. Therefore, the synergistic effect of these two drugs enhances the efficacy of Ku-Shen, aligning with the traditional Chinese medicine concept of compatibility. Analyzing the interaction and characteristic mechanisms of these drugs from various perspectives can improve guidance for clinical practice and enhance our modern understanding of TCM principles [28]. Consequently, this approach systematically and comprehensively elucidates the mode of action of each Chinese medicine in Chinese medicine prescription at the molecular level [29].

CKI is clinically recognized for its broad-spectrum anticancer activity and has shown effective therapeutic results across various types of cancer. However, the underlying therapeutic mechanisms and potential roles remain poorly understood. Yang [30] discovered that CKI can directly target and kill tumor cells or regulate the tumor immune microenvironment, thereby enhancing the efficacy of chemotherapy drugs. Furthermore, through a pathway analysis, it was found that CKI targets the EGFR-STAT3 signaling pathway, effectively inhibiting tumor cell growth, migration, and invasion while promoting apoptosis. Wang [31] investigated the active components in CKI and analyzed the pharmacological and anticancer effects of each component. While previous studies have explored the mechanisms of CKI, they did not directly examine the interactions between components and targets. Instead, they focused on classical anticancer signaling pathways, a process that is often labor-intensive and time-consuming. This study, however, directly examines the effects of drug components on target genes using a network analysis, allowing for more precise research. This approach provides a new method for studying the therapeutic mechanisms of CKI.

Based on a PPI network, we ranked each protein by its degree value, and selected the top three core proteins EGFR and ESR1 for potential targets for liver cancer. The experimental validation scores for these proteins, particularly EGFR, were relatively high (as shown in Table S1), providing a strong basis for subsequent experimental investigations. Additionally, we also observed that the selected core targets play a key regulatory role in the occurrence and development of tumors. For example, EGFR plays an important role in the growth and reproduction of tumor cells [32], and ESR1 can cause abnormalities in the estrogen signaling pathway, thereby affecting the growth and metastasis of tumor cells [33]. CKI primarily addresses liver cancer through the modulation of diverse pathways, such as neuroactive ligand–receptor interactions, the cAMP signaling pathway, various cancer-related pathways, and serotonergic synapse activity. The molecular docking experiments revealed that key active components of CKI, including matrine and oxymatrine, have a strong binding affinity for the three core targets, especially EGFR. Based on the re-docking results, all RMSD values were less than 2 Å, indicating that the molecular docking method and its parameter settings are reliable. This suggests a robust interaction between EGFR and oxymatrine. In vitro experiments further confirmed that these components effectively inhibit the proliferation of HepG2 cells. Additionally, oxymatrine was shown to regulate the expression of EGFR, a core therapeutic target, contributing to its efficacy in treating liver cancer [34]. We have validated the core target EGFR through WB experiments. However, based solely on WB experiments, we cannot yet determine the related pathways and other regulatory mechanisms. In future research, we will combine other experiments (such as co-immunoprecipitation and mass spectrometry) to further investigate the relevant regulatory mechanisms.

In conclusion, this study comprehensively and efficiently investigated the active components, targets, protein interaction networks, and biological pathways of CKI in treating liver cancer. It also clarified the internal compatibility of CKI from a network pharmacology perspective, and confirmed these findings through molecular docking and in vitro experiments. The results provide a valuable reference for future pharmacological research on CKI and its application in liver cancer treatment.

## Figures and Tables

**Table 1:** The parameters of the virtual box.

	x- Center	y- Center	z- Center	x-Dimension	y-Dimension	z-Dimension	Shape	Spacing
Isomatrine–EGFR	36.02	178.10	164.24	126	130	126	square	0.992
Isomatrine–ESR1	−36.79	−25.98	−47.32	126	126	120	square	1.0
Matrine–EGFR	36.02	178.10	164.24	126	126	126	square	0.45
Matrine–ESR1	−36.79	−25.98	−47.32	126	124	126	square	0.47
Oxymatrine–EGFR	36.02	178.10	164.24	126	126	126	square	0.93
Oxymatrine–ESR1	−36.79	−25.98	−47.32	126	142	126	square	1.0

**Table 2:** The active ingredients of CKI.

Ingredients	PubChem CID	
Piscidic Acid	120693	CKI 1
Macrozamin	9576780	CKI 2
Oxysophoridine	71773433	CKI 3
Oxymatrine	114850	CKI 4
Oxysophocarpine	24721085	CKI 5
Sophoridine	165549	CKI 6
Sophoranol	12442899	CKI 7
Baptifoline	621307	CKI 8
9a-Hydroxymatrine	15385684	CKI 9
N-methylcytisine	670971	CKI 10
Lamprolobine	87752	CKI 11
Isomatrine	5271984	CKI 12
Matrine	91466	CKI 13
Sophocarpine	115269	CKI 14
Trifolirhizin	442827	CKI 15
9α-Hydroxysophocarpine	132555388	CKI 16

**Table 3:** Topological parameters of PPI network for CKI.

Target	Degree	BC	Target	Degree	BC
HDAC1	20	295.966	HSD17B7	4.0	0.0
ESR1	16	543.3	CHRNA1	4.0	2.0
EGFR	14	546.0	BRD4	4.0	0.0
JAK2	14	364.0	ROCK2	2.0	0.0
CHRNA4	14	40.5	ROCK1	2.0	0.0
CHRNB2	14	40.5	PARP2	2.0	0.0
CHRNA3	14	40.5	ITGB3	2.0	0.0
CHRNB4	14	40.5	ITGA2B	2.0	0.0
HDAC2	12	25.8	IMPDH2	2.0	0.0
CHRNA7	12	0	IMPDH1	2.0	0.0
CHRNA9	12	0	HDAC10	2.0	0.0
CHRNA2	12	0	HCN4	2.0	0.0
KDM1A	10	11.167	HCN1	2.0	0.0
AR	10	24.8	FEN1	2.0	0.0
HDAC3	10	0.8	PRKCA	2.0	0.0
PARP1	10	126.0	HDAC6	2.0	0.0
JAK1	6	0	CYP2C9	2.0	0.0
HSPA8	6	280.0	CYP2C19	2.0	0.0
ERG28	6.0	31.0	CTSL	2.0	0.0
SIGMAR1	6.0	180.0	CTSK	2.0	0.0
CYP51A1	6.0	31.0	CHRNG	2.0	0.0
CIR1	6.0	0	CHRND	2.0	0.0
HSPA5		232.0	BAZ2A	2.0	0.0
HSPA5	4.0	232.0	ADRA1B	2.0	0.0
ESR2	4.0	0.0	ADRA1A	2.0	0.0

Note: BC represents betweenness centrality.

**Table 4:** Molecular docking results for active ingredients of CKI and their target sites.

Ingredient	Minimum Binding Energy (kcal/mol)	
	EGFR	ESR1
Matrine	−5.5	−5.5
Oxymatrine	−9.5	−7.4
Isomatrine	−7.5	−6.3

## Data Availability

The datasets presented in this study can be found in online repositories. The names of the repository/repositories and accession number(s) can be found in the article/Supplementary Materials.

## Supplementary Materials

The following supporting information can be downloaded at: https://www.mdpi.com/article/10.3390/genes15101278/s1, File S1: Protein node interaction information; File S2: Details of re-docking. File S3: Details of molecular docking.

## Abbreviations

TCM	Traditional Chinese Medicine
CKI	Compound Ku-Shen Injection
TCMSP	The Chinese Medicine System Pharmacology Database
OB	Oral Bioavailability
DL	Drug Similarity
PPI	Protein–Protein Interaction
GO	Gene Ontology
KEGG	Kyoto Encyclopedia of Genes and Genomes
BP	Biological Process
CC	Cellular Component
MF	Molecular Function
EGFR	Epidermal Growth Factor Receptor
p-EGFR	Phosphorylated EGFR
ESR1	Estrogen Receptor 1
HDAC1	Histone Deacetylase 1
IC50	The 50% Inhibitory Concentration
RMSD	Root Mean Square Deviation
BC	Betweenness Centrality
