# Supplementary material for: Unveiling the Mechanism of Compound Ku-Shen Injection in Liver Cancer Treatment through an Ingredient–Target Network Analysis
Source: Genes (Basel). 2024 Sep 29;15(10):1278. doi: 10.3390/genes15101278 (PMC11507192; doi:10.3390/genes15101278)
Supplement: Supplementary file 1 [file genes-15-01278-s001.zip › S1/S1.pdf]

#node1	node2	node1_stri	node2_str	neighborhc	gene_fusic	phylogenet	homology	coexpressi	experiment	database_a	automated_	combined_s
ACE	REN	9606.ENSP(	9606.ENSP(	0	0	0	0	0.044	0	0	0.987	0.987
ACE	AGTR1	9606.ENSP(	9606.ENSP(	0	0	0	0	0.042	0	0	0.974	0.974
ACHE	CHKA	9606.ENSP(	9606.ENSP(	0	0	0	0	0	0	0.9	0.158	0.912
ADH1A	ADH1C	9606.ENSP(	9606.ENSP(	0	0	0.048	0.985	0.777	0	0.9	0.302	0.983
ADH1C	ADH1A	9606.ENSP(	9606.ENSP(	0	0	0.048	0.985	0.777	0	0.9	0.302	0.983
ADK	PDE4D	9606.ENSP(	9606.ENSP(	0	0	0	0	0	0	0.9	0.269	0.923
ADK	PDE7A	9606.ENSP(	9606.ENSP(	0	0	0	0	0	0	0.9	0.29	0.925
ADORA1	ADORA3	9606.ENSP(	9606.ENSP(	0	0	0.053	0.935	0	0	0.9	0.101	0.907
ADORA3	ADORA1	9606.ENSP(	9606.ENSP(	0	0	0.053	0.935	0	0	0.9	0.101	0.907
ADRA1A	ADRA1D	9606.ENSP(	9606.ENSP(	0	0	0	0.929	0.078	0	0.9	0.093	0.909
ADRA1D	ADRA1A	9606.ENSP(	9606.ENSP(	0	0	0	0.929	0.078	0	0.9	0.093	0.909
ADRB1	ADRB3	9606.ENSP(	9606.ENSP(	0	0	0	0.929	0.051	0	0.9	0.106	0.907
ADRB2	ADRB3	9606.ENSP(	9606.ENSP(	0	0	0	0.916	0.051	0	0.9	0.426	0.94
ADRB3	ADRB2	9606.ENSP(	9606.ENSP(	0	0	0	0.916	0.051	0	0.9	0.426	0.94
ADRB3	ADRB1	9606.ENSP(	9606.ENSP(	0	0	0	0.929	0.051	0	0.9	0.106	0.907
AGTR1	REN	9606.ENSP(	9606.ENSP(	0	0	0	0	0	0	0	0.969	0.969
AGTR1	ACE	9606.ENSP(	9606.ENSP(	0	0	0	0	0.042	0	0	0.974	0.974
ALK	JAK1	9606.ENSP(	9606.ENSP(	0	0	0	0.561	0	0	0.9	0.465	0.944
AMY2A	GAA	9606.ENSP(	9606.ENSP(	0.056	0	0	0	0.067	0	0.907	0.529	0.956
AMY2A	MGAM	9606.ENSP(	9606.ENSP(	0.056	0	0	0	0.067	0	0.907	0.988	0.998
AR	ESR1	9606.ENSP(	9606.ENSP(	0	0	0	0.651	0.172	0	0	0.978	0.981
CHKA	ACHE	9606.ENSP(	9606.ENSP(	0	0	0	0	0	0	0.9	0.158	0.912
CHRM1	CHRM3	9606.ENSP(	9606.ENSP(	0	0	0.055	0.949	0.09	0	0.9	0.083	0.91
CHRM1	GRM5	9606.ENSP(	9606.ENSP(	0	0	0	0	0.255	0	0.9	0.389	0.95
CHRM1	CHRM5	9606.ENSP(	9606.ENSP(	0	0	0.053	0.947	0.042	0	0.9	0.08	0.905
CHRM1	CHRM2	9606.ENSP(	9606.ENSP(	0	0	0.059	0.921	0.053	0	0.9	0.095	0.908
CHRM2	CHRM1	9606.ENSP(	9606.ENSP(	0	0	0.059	0.921	0.053	0	0.9	0.095	0.908
CHRM3	CHRM1	9606.ENSP(	9606.ENSP(	0	0	0.055	0.949	0.09	0	0.9	0.083	0.91
CHRM3	GRM5	9606.ENSP(	9606.ENSP(	0	0	0	0	0.097	0	0.9	0.281	0.929
CHRM5	GRM5	9606.ENSP(	9606.ENSP(	0	0	0	0	0.07	0	0.9	0.47	0.946
CHRM5	CHRM1	9606.ENSP(	9606.ENSP(	0	0	0.053	0.947	0.042	0	0.9	0.08	0.905
CHRNA2	CHRNA4	9606.ENSP(	9606.ENSP(	0	0	0.058	0.961	0.072	0	0.9	0.391	0.939
CHRNA4	CHRNA2	9606.ENSP(	9606.ENSP(	0	0	0.058	0.961	0.072	0	0.9	0.391	0.939
CPA1	CPA3	9606.ENSP(	9606.ENSP(	0	0	0.098	0.884	0	0	0.9	0.207	0.922
CPA3	CPA1	9606.ENSP(	9606.ENSP(	0	0	0.098	0.884	0	0	0.9	0.207	0.922
CYP19A1	SRD5A1	9606.ENSP(	9606.ENSP(	0	0	0	0	0	0	0.9	0.732	0.972
CYP19A1	SRD5A2	9606.ENSP(	9606.ENSP(	0	0	0	0	0	0	0.9	0.627	0.961
CYP2C19	PTGS2	9606.ENSP(	9606.ENSP(	0	0	0	0	0.056	0	0.9	0.293	0.927
CYP2C19	CYP2D6	9606.ENSP(	9606.ENSP(	0	0	0.096	0.888	0.074	0	0.907	0.147	0.925
CYP2C9	PTGS2	9606.ENSP(	9606.ENSP(	0	0	0	0	0.056	0	0.9	0.377	0.936
CYP2D6	CYP2C19	9606.ENSP(	9606.ENSP(	0	0	0.096	0.888	0.074	0	0.907	0.147	0.925
DPP4	SLC5A2	9606.ENSP(	9606.ENSP(	0	0	0	0	0.042	0	0	0.909	0.909
DPP4	MME	9606.ENSP(	9606.ENSP(	0	0	0	0	0.073	0	0.9	0.624	0.962
DRD1	SLC6A3	9606.ENSP(	9606.ENSP(	0	0	0	0	0	0	0	0.906	0.906
DRD2	SLC6A4	9606.ENSP(	9606.ENSP(	0	0	0	0	0.054	0	0	0.908	0.91
DRD3	DRD4	9606.ENSP(	9606.ENSP(	0	0	0	0.864	0.056	0	0.9	0.454	0.943
DRD4	DRD3	9606.ENSP(	9606.ENSP(	0	0	0	0.864	0.056	0	0.9	0.454	0.943
DRD4	SLC6A3	9606.ENSP(	9606.ENSP(	0	0	0	0	0.054	0	0	0.96	0.96
DRD4	SLC6A4	9606.ENSP(	9606.ENSP(	0	0	0	0	0.054	0	0	0.978	0.978
EPHX2	PNP	9606.ENSP(	9606.ENSP(	0.076	0	0	0	0.122	0	0.826	0.546	0.927
ERCC5	KDM1A	9606.ENSP(	9606.ENSP(	0	0	0	0	0.049	0	0.9	0	0.9
ESR1	AR	9606.ENSP(	9606.ENSP(	0	0	0	0.651	0.172	0	0	0.978	0.981
GAA	MGAM	9606.ENSP(	9606.ENSP(	0	0	0.086	0.901	0	0	0.9	0.42	0.942
GAA	AMY2A	9606.ENSP(	9606.ENSP(	0.056	0	0	0	0.067	0	0.907	0.529	0.956
GBA2	UGCG	9606.ENSP(	9606.ENSP(	0	0	0	0	0	0	0.9	0.762	0.975
GRM5	CHRM3	9606.ENSP(	9606.ENSP(	0	0	0	0	0.097	0	0.9	0.281	0.929
GRM5	CHRM5	9606.ENSP(	9606.ENSP(	0	0	0	0	0.07	0	0.9	0.47	0.946
GRM5	CHRM1	9606.ENSP(	9606.ENSP(	0	0	0	0	0.255	0	0.9	0.389	0.95
HDAC1	HDAC3	9606.ENSP(	9606.ENSP(	0	0	0.059	0.963	0.262	0	0.9	0.058	0.925
HDAC2	HDAC3	9606.ENSP(	9606.ENSP(	0	0	0.059	0.963	0.067	0	0.9	0.056	0.906
HDAC3	HDAC2	9606.ENSP(	9606.ENSP(	0	0	0.059	0.963	0.067	0	0.9	0.056	0.906
HDAC3	HDAC1	9606.ENSP(	9606.ENSP(	0	0	0.059	0.963	0.262	0	0.9	0.058	0.925
HSD17B3	SRD5A1	9606.ENSP(	9606.ENSP(	0	0	0	0	0.069	0	0.9	0.791	0.978
HSD17B3	SRD5A2	9606.ENSP(	9606.ENSP(	0	0	0	0	0.077	0	0.9	0.799	0.979
HTR1A	SLC6A4	9606.ENSP(	9606.ENSP(	0	0	0	0	0.054	0	0	0.968	0.968
HTR1B	SLC6A4	9606.ENSP(	9606.ENSP(	0	0	0	0	0.054	0	0	0.931	0.932
HTR1D	SLC6A4	9606.ENSP(	9606.ENSP(	0	0	0	0	0.054	0	0	0.92	0.921
HTR2C	SLC6A4	9606.ENSP(	9606.ENSP(	0	0	0	0	0	0	0	0.914	0.914
HTR6	HTR7	9606.ENSP(	9606.ENSP(	0	0	0	0.741	0	0	0.9	0.288	0.925
HTR7	HTR6	9606.ENSP(	9606.ENSP(	0	0	0	0.741	0	0	0.9	0.288	0.925
IL2	JAK1	9606.ENSP(	9606.ENSP(	0	0	0	0	0	0	0.9	0.898	0.989
IL2	JAK3	9606.ENSP(	9606.ENSP(	0	0	0	0	0.042	0	0.9	0.996	0.999
JAK1	IL2	9606.ENSP(	9606.ENSP(	0	0	0	0	0	0	0.9	0.898	0.989
JAK1	ALK	9606.ENSP(	9606.ENSP(	0	0	0	0.561	0	0	0.9	0.465	0.944
JAK3	IL2	9606.ENSP(	9606.ENSP(	0	0	0	0	0.042	0	0.9	0.996	0.999

KDM1A	ERCC5	9606.ENSP(	9606.ENSP(	0	0	0	0	0.049	0	0.9	0	0.9
MGAM	GAA	9606.ENSP(	9606.ENSP(	0	0	0.086	0.901	0	0	0.9	0.42	0.942
MGAM	AMY2A	9606.ENSP(	9606.ENSP(	0.056	0	0	0	0.067	0	0.907	0.988	0.998
MME	DPP4	9606.ENSP(	9606.ENSP(	0	0	0	0	0.073	0	0.9	0.624	0.962
MMP1	MMP3	9606.ENSP(	9606.ENSP(	0	0	0.064	0.951	0.657	0	0.9	0.597	0.985
MMP1	MMP9	9606.ENSP(	9606.ENSP(	0	0	0	0.743	0.154	0	0.9	0.725	0.974
MMP3	MMP9	9606.ENSP(	9606.ENSP(	0	0	0.075	0.784	0.145	0	0.9	0.514	0.956
MMP3	MMP1	9606.ENSP(	9606.ENSP(	0	0	0.064	0.951	0.657	0	0.9	0.597	0.985
MMP9	MPO	9606.ENSP(	9606.ENSP(	0	0	0	0	0.085	0	0	0.967	0.969
MMP9	MMP3	9606.ENSP(	9606.ENSP(	0	0	0.075	0.784	0.145	0	0.9	0.514	0.956
MMP9	MMP1	9606.ENSP(	9606.ENSP(	0	0	0	0.743	0.154	0	0.9	0.725	0.974
MPO	MMP9	9606.ENSP(	9606.ENSP(	0	0	0	0	0.085	0	0	0.967	0.969
PDE4D	ADK	9606.ENSP(	9606.ENSP(	0	0	0	0	0	0	0.9	0.269	0.923
PDE7A	ADK	9606.ENSP(	9606.ENSP(	0	0	0	0	0	0	0.9	0.29	0.925
PLA2G10	PTGS2	9606.ENSP(	9606.ENSP(	0	0	0	0	0	0	0.65	0.801	0.927
PNP	EPHX2	9606.ENSP(	9606.ENSP(	0.076	0	0	0	0.122	0	0.826	0.546	0.927
PRKCA	SLC6A3	9606.ENSP(	9606.ENSP(	0	0	0	0	0	0	0.9	0.174	0.913
PTGER2	PTGER4	9606.ENSP(	9606.ENSP(	0	0	0	0.786	0.149	0	0.9	0.594	0.962
PTGER4	PTGER2	9606.ENSP(	9606.ENSP(	0	0	0	0.786	0.149	0	0.9	0.594	0.962
PTGS2	CYP2C9	9606.ENSP(	9606.ENSP(	0	0	0	0	0.056	0	0.9	0.377	0.936
PTGS2	PLA2G10	9606.ENSP(	9606.ENSP(	0	0	0	0	0	0	0.65	0.801	0.927
PTGS2	CYP2C19	9606.ENSP(	9606.ENSP(	0	0	0	0	0.056	0	0.9	0.293	0.927
REN	AGTR1	9606.ENSP(	9606.ENSP(	0	0	0	0	0	0	0	0.969	0.969
REN	ACE	9606.ENSP(	9606.ENSP(	0	0	0	0	0.044	0	0	0.987	0.987
SLC18A2	SLC18A3	9606.ENSP(	9606.ENSP(	0	0	0.074	0.87	0	0	0.9	0.138	0.913
SLC18A3	SLC18A2	9606.ENSP(	9606.ENSP(	0	0	0.074	0.87	0	0	0.9	0.138	0.913
SLC5A2	DPP4	9606.ENSP(	9606.ENSP(	0	0	0	0	0.042	0	0	0.909	0.909
SLC6A3	DRD4	9606.ENSP(	9606.ENSP(	0	0	0	0	0.054	0	0	0.96	0.96
SLC6A3	DRD1	9606.ENSP(	9606.ENSP(	0	0	0	0	0	0	0	0.906	0.906
SLC6A3	PRKCA	9606.ENSP(	9606.ENSP(	0	0	0	0	0	0	0.9	0.174	0.913
SLC6A4	DRD4	9606.ENSP(	9606.ENSP(	0	0	0	0	0.054	0	0	0.978	0.978
SLC6A4	DRD2	9606.ENSP(	9606.ENSP(	0	0	0	0	0.054	0	0	0.908	0.91
SLC6A4	HTR2C	9606.ENSP(	9606.ENSP(	0	0	0	0	0	0	0	0.914	0.914
SLC6A4	HTR1D	9606.ENSP(	9606.ENSP(	0	0	0	0	0.054	0	0	0.92	0.921
SLC6A4	HTR1B	9606.ENSP(	9606.ENSP(	0	0	0	0	0.054	0	0	0.931	0.932
SLC6A4	HTR1A	9606.ENSP(	9606.ENSP(	0	0	0	0	0.054	0	0	0.968	0.968
SRD5A1	SRD5A2	9606.ENSP(	9606.ENSP(	0	0	0.077	0.927	0	0	0.9	0.355	0.935
SRD5A1	CYP19A1	9606.ENSP(	9606.ENSP(	0	0	0	0	0	0	0.9	0.732	0.972
SRD5A1	HSD17B3	9606.ENSP(	9606.ENSP(	0	0	0	0	0.069	0	0.9	0.791	0.978
SRD5A2	SRD5A1	9606.ENSP(	9606.ENSP(	0	0	0.077	0.927	0	0	0.9	0.355	0.935
SRD5A2	HSD17B3	9606.ENSP(	9606.ENSP(	0	0	0	0	0.077	0	0.9	0.799	0.979
SRD5A2	CYP19A1	9606.ENSP(	9606.ENSP(	0	0	0	0	0	0	0.9	0.627	0.961
UGCG	GBA2	9606.ENSP(	9606.ENSP(	0	0	0	0	0	0	0.9	0.762	0.975
CHRNA3	JAK2	9606.ENSP(	9606.ENSP(	0	0	0	0	0	0.043	0.9	0.061	0.902
CHRNA4	JAK2	9606.ENSP(	9606.ENSP(	0	0	0	0	0	0.043	0.9	0.073	0.903
CHRNB2	JAK2	9606.ENSP(	9606.ENSP(	0	0	0	0	0	0.043	0.9	0.046	0.9
CHRNB4	JAK2	9606.ENSP(	9606.ENSP(	0	0	0	0	0	0.043	0.9	0.046	0.9
JAK2	CHRNB4	9606.ENSP(	9606.ENSP(	0	0	0	0	0	0.043	0.9	0.046	0.9
JAK2	CHRNA3	9606.ENSP(	9606.ENSP(	0	0	0	0	0	0.043	0.9	0.061	0.902
JAK2	CHRNB2	9606.ENSP(	9606.ENSP(	0	0	0	0	0	0.043	0.9	0.046	0.9
JAK2	CHRNA4	9606.ENSP(	9606.ENSP(	0	0	0	0	0	0.043	0.9	0.073	0.903
ADK	PNP	9606.ENSP(	9606.ENSP(	0.095	0	0	0	0.047	0.051	0.938	0.77	0.986
PNP	ADK	9606.ENSP(	9606.ENSP(	0.095	0	0	0	0.047	0.051	0.938	0.77	0.986
CYP19A1	ESR2	9606.ENSP(	9606.ENSP(	0	0	0	0	0.048	0.056	0	0.923	0.925
CYP19A1	ESR1	9606.ENSP(	9606.ENSP(	0	0	0	0	0.042	0.056	0	0.925	0.927
ESR1	CYP19A1	9606.ENSP(	9606.ENSP(	0	0	0	0	0.042	0.056	0	0.925	0.927
ESR2	CYP19A1	9606.ENSP(	9606.ENSP(	0	0	0	0	0.048	0.056	0	0.923	0.925
HCN4	SCN5A	9606.ENSP(	9606.ENSP(	0	0	0	0	0.088	0.058	0	0.949	0.953
SCN5A	HCN4	9606.ENSP(	9606.ENSP(	0	0	0	0	0.088	0.058	0	0.949	0.953
KCNH2	SCN5A	9606.ENSP(	9606.ENSP(	0	0	0	0	0.048	0.059	0	0.978	0.978
SCN5A	KCNH2	9606.ENSP(	9606.ENSP(	0	0	0	0	0.048	0.059	0	0.978	0.978
CYP2C9	EPHX2	9606.ENSP(	9606.ENSP(	0.059	0	0	0	0.063	0.068	0.906	0.645	0.967
EPHX2	CYP2C9	9606.ENSP(	9606.ENSP(	0.059	0	0	0	0.063	0.068	0.906	0.645	0.967
CYP2C19	EPHX1	9606.ENSP(	9606.ENSP(	0	0	0	0	0.062	0.071	0.9	0.519	0.952
CYP2C9	EPHX1	9606.ENSP(	9606.ENSP(	0	0	0	0	0.062	0.071	0.9	0.629	0.963
EPHX1	CYP2C9	9606.ENSP(	9606.ENSP(	0	0	0	0	0.062	0.071	0.9	0.629	0.963
EPHX1	CYP2C19	9606.ENSP(	9606.ENSP(	0	0	0	0	0.062	0.071	0.9	0.519	0.952
CHRNA2	CHRNA7	9606.ENSP(	9606.ENSP(	0	0	0.087	0.879	0.086	0.091	0.9	0.229	0.93
CHRNA4	CHRNA7	9606.ENSP(	9606.ENSP(	0	0	0.083	0.853	0.086	0.091	0.9	0.172	0.925
CHRNA7	CHRNA4	9606.ENSP(	9606.ENSP(	0	0	0.083	0.853	0.086	0.091	0.9	0.172	0.925
CHRNA7	CHRNA2	9606.ENSP(	9606.ENSP(	0	0	0.087	0.879	0.086	0.091	0.9	0.229	0.93
CHRNA3	CHRNA7	9606.ENSP(	9606.ENSP(	0	0	0.067	0.884	0.092	0.092	0.9	0.105	0.918
CHRNA7	CHRNA3	9606.ENSP(	9606.ENSP(	0	0	0.067	0.884	0.092	0.092	0.9	0.105	0.918
CHRNB2	CHRNB4	9606.ENSP(	9606.ENSP(	0	0	0.058	0.963	0.042	0.095	0.9	0.073	0.91
CHRNB4	CHRNB2	9606.ENSP(	9606.ENSP(	0	0	0.058	0.963	0.042	0.095	0.9	0.073	0.91
CTSB	CTSL	9606.ENSP(	9606.ENSP(	0	0	0.129	0.648	0.212	0.095	0.9	0.647	0.974

CTSL	CTSB	9606.ENSP(	9606.ENSP(	0	0	0.129	0.648	0.212	0.095	0.9	0.647	0.974
CYP2D6	HSD11B1	9606.ENSP(	9606.ENSP(	0	0	0	0	0.091	0.095	0.911	0.121	0.927
HSD11B1	CYP2D6	9606.ENSP(	9606.ENSP(	0	0	0	0	0.091	0.095	0.911	0.121	0.927
CHRNA7	CHRNB4	9606.ENSP(	9606.ENSP(	0	0	0.071	0.874	0.134	0.1	0.9	0.118	0.924
CHRNB4	CHRNA7	9606.ENSP(	9606.ENSP(	0	0	0.071	0.874	0.134	0.1	0.9	0.118	0.924
CHRNA7	CHRNB2	9606.ENSP(	9606.ENSP(	0	0	0.081	0.863	0.104	0.109	0.9	0.119	0.923
CHRNB2	CHRNA7	9606.ENSP(	9606.ENSP(	0	0	0.081	0.863	0.104	0.109	0.9	0.119	0.923
HSPA5	SIGMAR1	9606.ENSP(	9606.ENSP(	0	0	0	0	0.042	0.113	0	0.992	0.992
SIGMAR1	HSPA5	9606.ENSP(	9606.ENSP(	0	0	0	0	0.042	0.113	0	0.992	0.992
CHRNA2	CHRNB4	9606.ENSP(	9606.ENSP(	0	0	0.074	0.927	0.058	0.117	0.9	0.104	0.918
CHRNA2	CHRNA3	9606.ENSP(	9606.ENSP(	0	0	0.064	0.95	0.051	0.117	0.9	0.083	0.915
CHRNA2	CHRNB2	9606.ENSP(	9606.ENSP(	0	0	0.07	0.93	0.085	0.117	0.9	0.418	0.948
CHRNA3	CHRNA2	9606.ENSP(	9606.ENSP(	0	0	0.064	0.95	0.051	0.117	0.9	0.083	0.915
CHRNA3	CHRNA4	9606.ENSP(	9606.ENSP(	0	0	0.066	0.945	0.044	0.117	0.9	0.091	0.915
CHRNA4	CHRNA3	9606.ENSP(	9606.ENSP(	0	0	0.066	0.945	0.044	0.117	0.9	0.091	0.915
CHRNB2	CHRNA2	9606.ENSP(	9606.ENSP(	0	0	0.07	0.93	0.085	0.117	0.9	0.418	0.948
CHRNB4	CHRNA2	9606.ENSP(	9606.ENSP(	0	0	0.074	0.927	0.058	0.117	0.9	0.104	0.918
CHRNA4	CHRNB4	9606.ENSP(	9606.ENSP(	0	0	0.08	0.915	0.051	0.126	0.9	0.118	0.92
CHRNB4	CHRNA4	9606.ENSP(	9606.ENSP(	0	0	0.08	0.915	0.051	0.126	0.9	0.118	0.92
CYP19A1	HSD17B3	9606.ENSP(	9606.ENSP(	0	0	0	0	0.109	0.129	0.906	0.66	0.972
HSD17B3	CYP19A1	9606.ENSP(	9606.ENSP(	0	0	0	0	0.109	0.129	0.906	0.66	0.972
CHRNA2	CHRNB3	9606.ENSP(	9606.ENSP(	0	0	0.067	0.945	0.072	0.137	0.9	0.085	0.919
CHRNB3	CHRNA2	9606.ENSP(	9606.ENSP(	0	0	0.067	0.945	0.072	0.137	0.9	0.085	0.919
SLC22A2	SLC47A1	9606.ENSP(	9606.ENSP(	0	0	0	0	0.067	0.145	0	0.887	0.902
SLC47A1	SLC22A2	9606.ENSP(	9606.ENSP(	0	0	0	0	0.067	0.145	0	0.887	0.902
EGFR	JAK3	9606.ENSP(	9606.ENSP(	0	0	0	0.583	0	0.161	0.9	0.522	0.956
JAK3	EGFR	9606.ENSP(	9606.ENSP(	0	0	0	0.583	0	0.161	0.9	0.522	0.956
ALK	JAK2	9606.ENSP(	9606.ENSP(	0	0	0	0.562	0	0.162	0.9	0.307	0.936
JAK2	ALK	9606.ENSP(	9606.ENSP(	0	0	0	0.562	0	0.162	0.9	0.307	0.936
CHRNA5	CHRNB4	9606.ENSP(	9606.ENSP(	0	0	0.076	0.913	0.077	0.212	0.8	0.491	0.919
CHRNB4	CHRNA5	9606.ENSP(	9606.ENSP(	0	0	0.076	0.913	0.077	0.212	0.8	0.491	0.919
ADRB1	ADRB2	9606.ENSP(	9606.ENSP(	0	0	0	0.935	0.049	0.237	0.9	0.469	0.956
ADRB2	ADRB1	9606.ENSP(	9606.ENSP(	0	0	0	0.935	0.049	0.237	0.9	0.469	0.956
CYP19A1	HSD17B7	9606.ENSP(	9606.ENSP(	0	0	0	0	0	0.286	0.9	0.5	0.961
HSD17B7	CYP19A1	9606.ENSP(	9606.ENSP(	0	0	0	0	0	0.286	0.9	0.5	0.961
ADRA1B	ADRA1D	9606.ENSP(	9606.ENSP(	0	0	0	0.93	0.099	0.292	0.9	0.301	0.949
ADRA1D	ADRA1B	9606.ENSP(	9606.ENSP(	0	0	0	0.93	0.099	0.292	0.9	0.301	0.949
EGFR	SLC5A1	9606.ENSP(	9606.ENSP(	0	0	0	0	0.056	0.292	0	0.938	0.954
HTR1B	HTR1D	9606.ENSP(	9606.ENSP(	0	0	0.057	0.963	0.078	0.292	0.4	0.891	0.952
HTR1D	HTR1B	9606.ENSP(	9606.ENSP(	0	0	0.057	0.963	0.078	0.292	0.4	0.891	0.952
JAK1	TYK2	9606.ENSP(	9606.ENSP(	0	0	0.07	0.923	0.049	0.292	0.9	0.983	0.998
JAK2	JAK3	9606.ENSP(	9606.ENSP(	0	0	0.057	0.941	0.045	0.292	0.9	0.681	0.975
JAK2	TYK2	9606.ENSP(	9606.ENSP(	0	0	0.071	0.877	0.089	0.292	0.8	0.982	0.997
JAK3	JAK2	9606.ENSP(	9606.ENSP(	0	0	0.057	0.941	0.045	0.292	0.9	0.681	0.975
JAK3	TYK2	9606.ENSP(	9606.ENSP(	0	0	0.066	0.866	0.085	0.292	0	0.864	0.906
SLC5A1	EGFR	9606.ENSP(	9606.ENSP(	0	0	0	0	0.056	0.292	0	0.938	0.954
TYK2	JAK2	9606.ENSP(	9606.ENSP(	0	0	0.071	0.877	0.089	0.292	0.8	0.982	0.997
TYK2	JAK3	9606.ENSP(	9606.ENSP(	0	0	0.066	0.866	0.085	0.292	0	0.864	0.906
TYK2	JAK1	9606.ENSP(	9606.ENSP(	0	0	0.07	0.923	0.049	0.292	0.9	0.983	0.998
SLC18A2	SLC6A3	9606.ENSP(	9606.ENSP(	0	0	0	0	0.14	0.296	0	0.949	0.966
SLC6A3	SLC18A2	9606.ENSP(	9606.ENSP(	0	0	0	0	0.14	0.296	0	0.949	0.966
ADORA2A	GRM5	9606.ENSP(	9606.ENSP(	0	0	0	0	0	0.3	0	0.968	0.976
GRM5	ADORA2A	9606.ENSP(	9606.ENSP(	0	0	0	0	0	0.3	0	0.968	0.976
CHRM3	CHRM5	9606.ENSP(	9606.ENSP(	0	0	0.059	0.94	0	0.322	0.9	0.086	0.933
CHRM5	CHRM3	9606.ENSP(	9606.ENSP(	0	0	0.059	0.94	0	0.322	0.9	0.086	0.933
HTR1A	HTR1B	9606.ENSP(	9606.ENSP(	0	0	0	0.901	0.083	0.322	0	0.873	0.914
HTR1B	HTR1A	9606.ENSP(	9606.ENSP(	0	0	0	0.901	0.083	0.322	0	0.873	0.914
HTR2B	HTR2C	9606.ENSP(	9606.ENSP(	0	0	0	0.906	0	0.322	0.9	0.7	0.977
HTR2C	HTR2B	9606.ENSP(	9606.ENSP(	0	0	0	0.906	0	0.322	0.9	0.7	0.977
ADORA2A	ADORA2B	9606.ENSP(	9606.ENSP(	0	0	0	0.953	0	0.325	0.9	0.153	0.937
ADORA2B	ADORA2A	9606.ENSP(	9606.ENSP(	0	0	0	0.953	0	0.325	0.9	0.153	0.937
ESR1	KDM1A	9606.ENSP(	9606.ENSP(	0	0	0	0	0	0.338	0.5	0.895	0.962
KDM1A	ESR1	9606.ENSP(	9606.ENSP(	0	0	0	0	0	0.338	0.5	0.895	0.962
ESR1	HDAC2	9606.ENSP(	9606.ENSP(	0	0	0	0	0	0.343	0	0.858	0.903
HDAC2	ESR1	9606.ENSP(	9606.ENSP(	0	0	0	0	0	0.343	0	0.858	0.903
CHRNA3	CHRNA5	9606.ENSP(	9606.ENSP(	0	0	0.075	0.925	0.062	0.359	0.8	0.855	0.98
CHRNA5	CHRNA3	9606.ENSP(	9606.ENSP(	0	0	0.075	0.925	0.062	0.359	0.8	0.855	0.98
ALK	JAK3	9606.ENSP(	9606.ENSP(	0	0	0	0.56	0	0.369	0.9	0.282	0.95
JAK3	ALK	9606.ENSP(	9606.ENSP(	0	0	0	0.56	0	0.369	0.9	0.282	0.95
DRD2	SLC6A3	9606.ENSP(	9606.ENSP(	0	0	0	0	0.054	0.38	0.9	0.995	0.999
SLC6A3	DRD2	9606.ENSP(	9606.ENSP(	0	0	0	0	0.054	0.38	0.9	0.995	0.999
BAZ2A	HDAC1	9606.ENSP(	9606.ENSP(	0	0	0	0	0.049	0.406	0.5	0.897	0.967
HDAC1	BAZ2A	9606.ENSP(	9606.ENSP(	0	0	0	0	0.049	0.406	0.5	0.897	0.967
EGFR	JAK1	9606.ENSP(	9606.ENSP(	0	0	0	0.572	0.045	0.417	0.9	0.537	0.97
JAK1	EGFR	9606.ENSP(	9606.ENSP(	0	0	0	0.572	0.045	0.417	0.9	0.537	0.97
FEN1	PARP1	9606.ENSP(	9606.ENSP(	0	0	0	0	0.184	0.419	0.5	0.781	0.941

PARP1	FEN1	9606.ENSP(	9606.ENSP(	0	0	0	0	0.184	0.419	0.5	0.781	0.941
JAK1	JAK2	9606.ENSP(	9606.ENSP(	0	0	0	0.908	0.113	0.51	0.9	0.982	0.999
JAK1	JAK3	9606.ENSP(	9606.ENSP(	0	0	0	0.888	0.057	0.51	0.9	0.983	0.999
JAK2	JAK1	9606.ENSP(	9606.ENSP(	0	0	0	0.908	0.113	0.51	0.9	0.982	0.999
JAK3	JAK1	9606.ENSP(	9606.ENSP(	0	0	0	0.888	0.057	0.51	0.9	0.983	0.999
ADRA1A	ADRA1B	9606.ENSP(	9606.ENSP(	0	0	0.063	0.935	0.08	0.541	0.9	0.1	0.957
ADRA1B	ADRA1A	9606.ENSP(	9606.ENSP(	0	0	0.063	0.935	0.08	0.541	0.9	0.1	0.957
ESR2	NCOR2	9606.ENSP(	9606.ENSP(	0	0	0	0	0	0.549	0.5	0.626	0.908
NCOR2	ESR2	9606.ENSP(	9606.ENSP(	0	0	0	0	0	0.549	0.5	0.626	0.908
CHRNA1	CHRNG	9606.ENSP(	9606.ENSP(	0	0	0.089	0.815	0.206	0.554	0.54	0.58	0.926
CHRNG	CHRNA1	9606.ENSP(	9606.ENSP(	0	0	0.089	0.815	0.206	0.554	0.54	0.58	0.926
HDAC10	NCOR2	9606.ENSP(	9606.ENSP(	0	0	0	0	0.084	0.572	0.5	0.842	0.965
NCOR2	HDAC10	9606.ENSP(	9606.ENSP(	0	0	0	0	0.084	0.572	0.5	0.842	0.965
EGFR	ESR1	9606.ENSP(	9606.ENSP(	0	0	0	0	0	0.583	0	0.974	0.988
ESR1	EGFR	9606.ENSP(	9606.ENSP(	0	0	0	0	0	0.583	0	0.974	0.988
AR	BRD4	9606.ENSP(	9606.ENSP(	0	0	0	0	0	0.585	0	0.929	0.969
BRD4	AR	9606.ENSP(	9606.ENSP(	0	0	0	0	0	0.585	0	0.929	0.969
CHRNA3	CHRNB2	9606.ENSP(	9606.ENSP(	0	0	0.071	0.924	0.044	0.602	0.9	0.102	0.962
CHRNB2	CHRNA3	9606.ENSP(	9606.ENSP(	0	0	0.071	0.924	0.044	0.602	0.9	0.102	0.962
PARP1	PARP2	9606.ENSP(	9606.ENSP(	0	0	0.098	0.87	0.13	0.613	0.8	0.513	0.965
PARP2	PARP1	9606.ENSP(	9606.ENSP(	0	0	0.098	0.87	0.13	0.613	0.8	0.513	0.965
HCN1	HCN4	9606.ENSP(	9606.ENSP(	0	0	0.05	0.956	0.044	0.619	0.72	0.169	0.904
HCN4	HCN1	9606.ENSP(	9606.ENSP(	0	0	0.05	0.956	0.044	0.619	0.72	0.169	0.904
AR	KDM1A	9606.ENSP(	9606.ENSP(	0	0	0	0	0	0.632	0.5	0.982	0.996
KDM1A	AR	9606.ENSP(	9606.ENSP(	0	0	0	0	0	0.632	0.5	0.982	0.996
HDAC2	NCOR2	9606.ENSP(	9606.ENSP(	0	0	0	0	0.044	0.643	0.8	0.92	0.993
NCOR2	HDAC2	9606.ENSP(	9606.ENSP(	0	0	0	0	0.044	0.643	0.8	0.92	0.993
HDAC1	PARP1	9606.ENSP(	9606.ENSP(	0	0	0	0	0.124	0.647	0	0.701	0.9
PARP1	HDAC1	9606.ENSP(	9606.ENSP(	0	0	0	0	0.124	0.647	0	0.701	0.9
CYP2C19	CYP2C9	9606.ENSP(	9606.ENSP(	0	0	0.048	0.985	0.514	0.661	0.9	0.37	0.988
CYP2C9	CYP2C19	9606.ENSP(	9606.ENSP(	0	0	0.048	0.985	0.514	0.661	0.9	0.37	0.988
CHRNA1	CHRND	9606.ENSP(	9606.ENSP(	0	0	0.076	0.845	0.172	0.693	0.54	0.681	0.959
CHRND	CHRNA1	9606.ENSP(	9606.ENSP(	0	0	0.076	0.845	0.172	0.693	0.54	0.681	0.959
HDAC1	NCOR2	9606.ENSP(	9606.ENSP(	0	0	0	0	0.049	0.701	0.8	0.995	0.999
NCOR2	HDAC1	9606.ENSP(	9606.ENSP(	0	0	0	0	0.049	0.701	0.8	0.995	0.999
HDAC3	KDM1A	9606.ENSP(	9606.ENSP(	0	0	0	0	0.135	0.72	0.4	0.395	0.9
KDM1A	HDAC3	9606.ENSP(	9606.ENSP(	0	0	0	0	0.135	0.72	0.4	0.395	0.9
EGFR	JAK2	9606.ENSP(	9606.ENSP(	0	0	0	0.576	0	0.723	0.9	0.564	0.986
JAK2	EGFR	9606.ENSP(	9606.ENSP(	0	0	0	0.576	0	0.723	0.9	0.564	0.986
CTSK	CTSL	9606.ENSP(	9606.ENSP(	0	0	0.071	0.937	0.068	0.741	0.4	0.426	0.908
CTSL	CTSK	9606.ENSP(	9606.ENSP(	0	0	0.071	0.937	0.068	0.741	0.4	0.426	0.908
HSPA5	HSPA8	9606.ENSP(	9606.ENSP(	0	0	0.057	0.966	0.308	0.756	0.5	0.823	0.983
HSPA8	HSPA5	9606.ENSP(	9606.ENSP(	0	0	0.057	0.966	0.308	0.756	0.5	0.823	0.983
ESR1	NCOR2	9606.ENSP(	9606.ENSP(	0	0	0	0	0.044	0.77	0.5	0.944	0.993
NCOR2	ESR1	9606.ENSP(	9606.ENSP(	0	0	0	0	0.044	0.77	0.5	0.944	0.993
EGFR	HDAC6	9606.ENSP(	9606.ENSP(	0	0	0	0	0	0.781	0	0.645	0.919
HDAC6	EGFR	9606.ENSP(	9606.ENSP(	0	0	0	0	0	0.781	0	0.645	0.919
ROCK1	ROCK2	9606.ENSP(	9606.ENSP(	0	0	0.056	0.968	0.195	0.781	0.9	0.409	0.988
ROCK2	ROCK1	9606.ENSP(	9606.ENSP(	0	0	0.056	0.968	0.195	0.781	0.9	0.409	0.988
EGFR	PRKCA	9606.ENSP(	9606.ENSP(	0	0	0	0.562	0.049	0.788	0	0.693	0.932
PRKCA	EGFR	9606.ENSP(	9606.ENSP(	0	0	0	0.562	0.049	0.788	0	0.693	0.932
AR	HDAC1	9606.ENSP(	9606.ENSP(	0	0	0	0	0	0.789	0	0.835	0.964
HDAC1	AR	9606.ENSP(	9606.ENSP(	0	0	0	0	0	0.789	0	0.835	0.964
BRD4	HDAC1	9606.ENSP(	9606.ENSP(	0	0	0	0	0.098	0.791	0	0.775	0.954
HDAC1	BRD4	9606.ENSP(	9606.ENSP(	0	0	0	0	0.098	0.791	0	0.775	0.954
ESR1	HSPA8	9606.ENSP(	9606.ENSP(	0	0	0	0	0	0.799	0	0.617	0.919
HSPA8	ESR1	9606.ENSP(	9606.ENSP(	0	0	0	0	0	0.799	0	0.617	0.919
CHRNA4	CHRNB2	9606.ENSP(	9606.ENSP(	0	0	0.071	0.922	0.144	0.802	0.9	0.683	0.994
CHRNB2	CHRNA4	9606.ENSP(	9606.ENSP(	0	0	0.071	0.922	0.144	0.802	0.9	0.683	0.994
ADORA2A	DRD2	9606.ENSP(	9606.ENSP(	0	0	0	0.639	0.1	0.806	0	0.987	0.997
DRD2	ADORA2A	9606.ENSP(	9606.ENSP(	0	0	0	0.639	0.1	0.806	0	0.987	0.997
ESR1	ESR2	9606.ENSP(	9606.ENSP(	0	0	0.062	0.923	0.042	0.83	0.9	0.934	0.998
ESR2	ESR1	9606.ENSP(	9606.ENSP(	0	0	0.062	0.923	0.042	0.83	0.9	0.934	0.998
EGFR	HSPA8	9606.ENSP(	9606.ENSP(	0	0	0	0	0.061	0.832	0	0.608	0.932
ESR1	HDAC1	9606.ENSP(	9606.ENSP(	0	0	0	0	0	0.832	0.5	0.987	0.998
HDAC1	ESR1	9606.ENSP(	9606.ENSP(	0	0	0	0	0	0.832	0.5	0.987	0.998
HSPA8	EGFR	9606.ENSP(	9606.ENSP(	0	0	0	0	0.061	0.832	0	0.608	0.932
IMPDH1	IMPDH2	9606.ENSP(	9606.ENSP(	0	0	0.049	0.983	0.054	0.846	0.9	0.056	0.984
IMPDH2	IMPDH1	9606.ENSP(	9606.ENSP(	0	0	0.049	0.983	0.054	0.846	0.9	0.056	0.984
AR	NCOR2	9606.ENSP(	9606.ENSP(	0	0	0	0	0	0.848	0.5	0.659	0.971
NCOR2	AR	9606.ENSP(	9606.ENSP(	0	0	0	0	0	0.848	0.5	0.659	0.971
CHRNA3	CHRNB4	9606.ENSP(	9606.ENSP(	0	0	0.075	0.927	0.075	0.9	0.9	0.822	0.998
CHRNB4	CHRNA3	9606.ENSP(	9606.ENSP(	0	0	0.075	0.927	0.075	0.9	0.9	0.822	0.998
HDAC2	KDM1A	9606.ENSP(	9606.ENSP(	0	0	0	0	0.133	0.941	0.4	0.997	0.999
KDM1A	HDAC2	9606.ENSP(	9606.ENSP(	0	0	0	0	0.133	0.941	0.4	0.997	0.999
HDAC1	KDM1A	9606.ENSP(	9606.ENSP(	0	0	0	0	0.176	0.942	0.4	0.998	0.999

KDM1A	HDAC1	9606.ENSP(	9606.ENSP(	0	0	0	0	0.176	0.942	0.4	0.998	0.999
CYP51A1	SIGMAR1	9606.ENSP(	9606.ENSP(	0	0	0	0	0.145	0.954	0	0.66	0.985
CYP51A1	HSD17B7	9606.ENSP(	9606.ENSP(	0	0	0	0	0.534	0.954	0	0.852	0.996
HSD17B7	CYP51A1	9606.ENSP(	9606.ENSP(	0	0	0	0	0.534	0.954	0	0.852	0.996
SIGMAR1	CYP51A1	9606.ENSP(	9606.ENSP(	0	0	0	0	0.145	0.954	0	0.66	0.985
ITGA2B	ITGB3	9606.ENSP(	9606.ENSP(	0	0	0	0	0.618	0.991	0.9	0.999	0.999
ITGB3	ITGA2B	9606.ENSP(	9606.ENSP(	0	0	0	0	0.618	0.991	0.9	0.999	0.999
HDAC3	NCOR2	9606.ENSP(	9606.ENSP(	0	0	0	0	0.049	0.992	0.8	0.995	0.999
NCOR2	HDAC3	9606.ENSP(	9606.ENSP(	0	0	0	0	0.049	0.992	0.8	0.995	0.999
HDAC1	HDAC2	9606.ENSP(	9606.ENSP(	0	0	0.049	0.983	0.101	0.994	0.9	0.982	0.999
HDAC2	HDAC1	9606.ENSP(	9606.ENSP(	0	0	0.049	0.983	0.101	0.994	0.9	0.982	0.999

score
