# Supplementary material for: Unveiling the Mechanism of Compound Ku-Shen Injection in Liver Cancer Treatment through an Ingredient–Target Network Analysis
Source: Genes (Basel). 2024 Sep 29;15(10):1278. doi: 10.3390/genes15101278 (PMC11507192; doi:10.3390/genes15101278)
Supplement: Supplementary file 1 [file genes-15-01278-s001.zip › S2/re-docking/3DT3/RMSD for 3DT3.pdf]

The re-docking results of RMSD for 3DT3

**Name**	**Reference**	**RMSD (Å)**
3DT3 1	3DT3 11	1. 4068
3DT3 2	3DT3 11	1. 4744
3DT3 3	3DT3 11	1. 4694
3DT3 4	3DT3 11	1. 4566
3DT3 5	3DT3 11	1. 5090
3DT3 6	3DT3 11	1. 4499
3DT3 7	3DT3 11	1. 5051
3DT3 8	3DT3 11	1. 4377
3DT3 9	3DT3 11	1. 4034
3DT3 10	3DT3 11	1. 4510
3DT3 11	3DT3 11	0. 0000
