# Supplementary material for: Unveiling the Mechanism of Compound Ku-Shen Injection in Liver Cancer Treatment through an Ingredient–Target Network Analysis
Source: Genes (Basel). 2024 Sep 29;15(10):1278. doi: 10.3390/genes15101278 (PMC11507192; doi:10.3390/genes15101278)
Supplement: Supplementary file 1 [file genes-15-01278-s001.zip › S2/re-docking/5I6Z/RMSD for 5I6Z.pdf]

The re-docking results of RMSD for 5I6Z

**Name**	**Reference**	**RMSD (Å)**
5I6Z 1	5I6Z 11	1. 6135
5I6Z 2	5I6Z 11	1. 7046
5I6Z 3	5I6Z 11	2.4767
5I6Z 4	5I6Z 11	1. 4566
5I6Z 5	5I6Z 11	2.5862
5I6Z 6	5I6Z 11	2.6645
5I6Z 7	5I6Z 11	2.6785
5I6Z 8	5I6Z 11	2.8754
5I6Z 9	5I6Z 11	2.9025
5I6Z 10	5I6Z 11	3.0258
5I6Z 11	5I6Z 11	0. 0000
