# Supplementary figures and images for: Unveiling the Mechanism of Compound Ku-Shen Injection in Liver Cancer Treatment through an Ingredient–Target Network Analysis
Source: Genes (Basel). 2024 Sep 29;15(10):1278. doi: 10.3390/genes15101278 (PMC11507192; doi:10.3390/genes15101278)

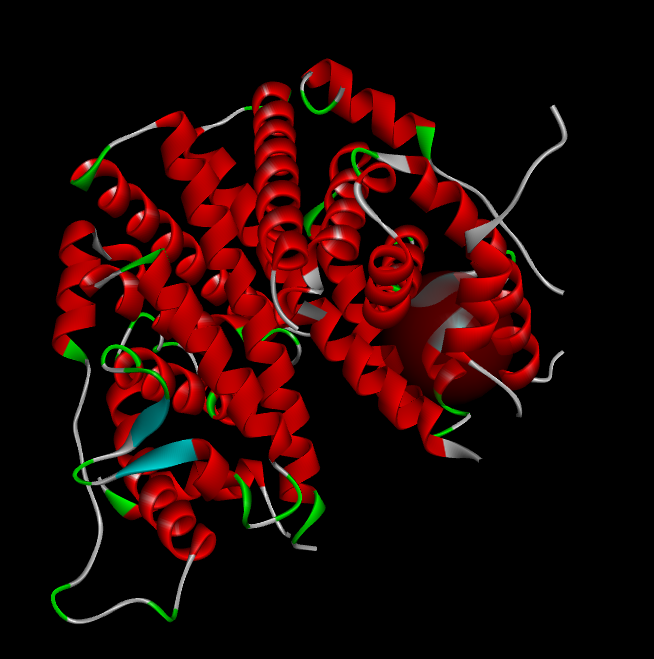

Supplement: Supplementary file 1 [file genes-15-01278-s001.zip › S2/re-docking/3DT3/3DT3.png]

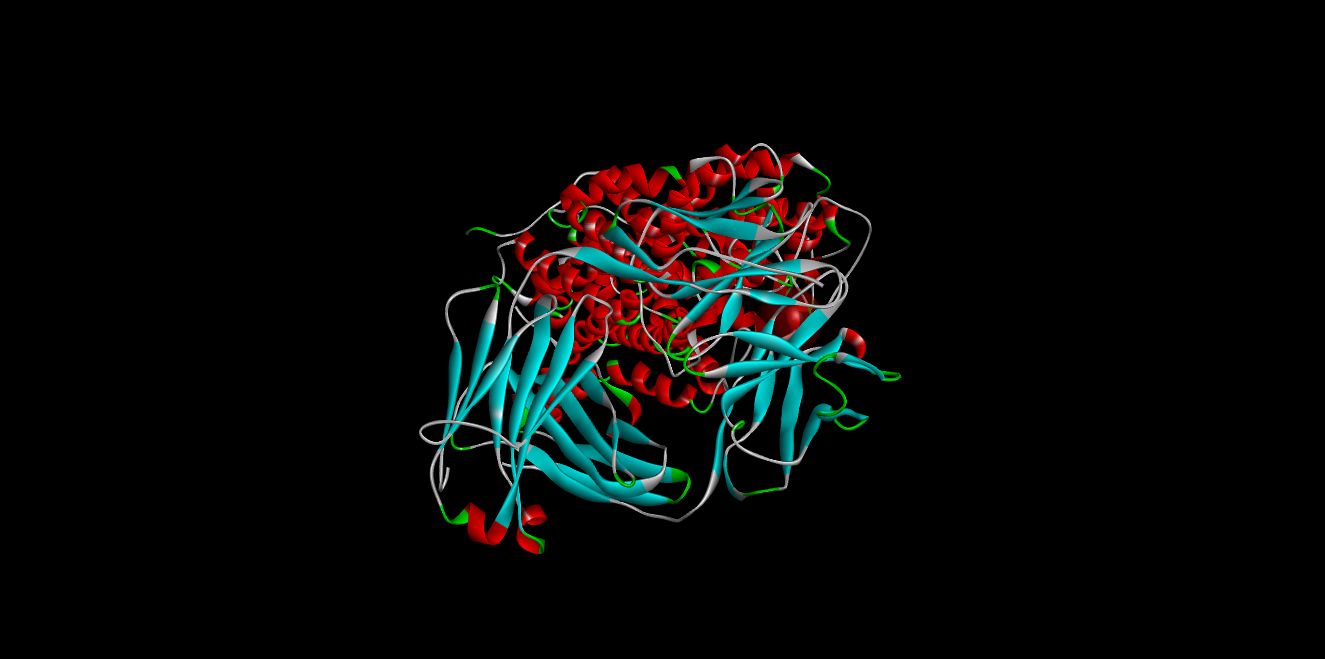

Supplement: Supplementary file 1 [file genes-15-01278-s001.zip › S2/re-docking/5I6Z/5I6Z.png]

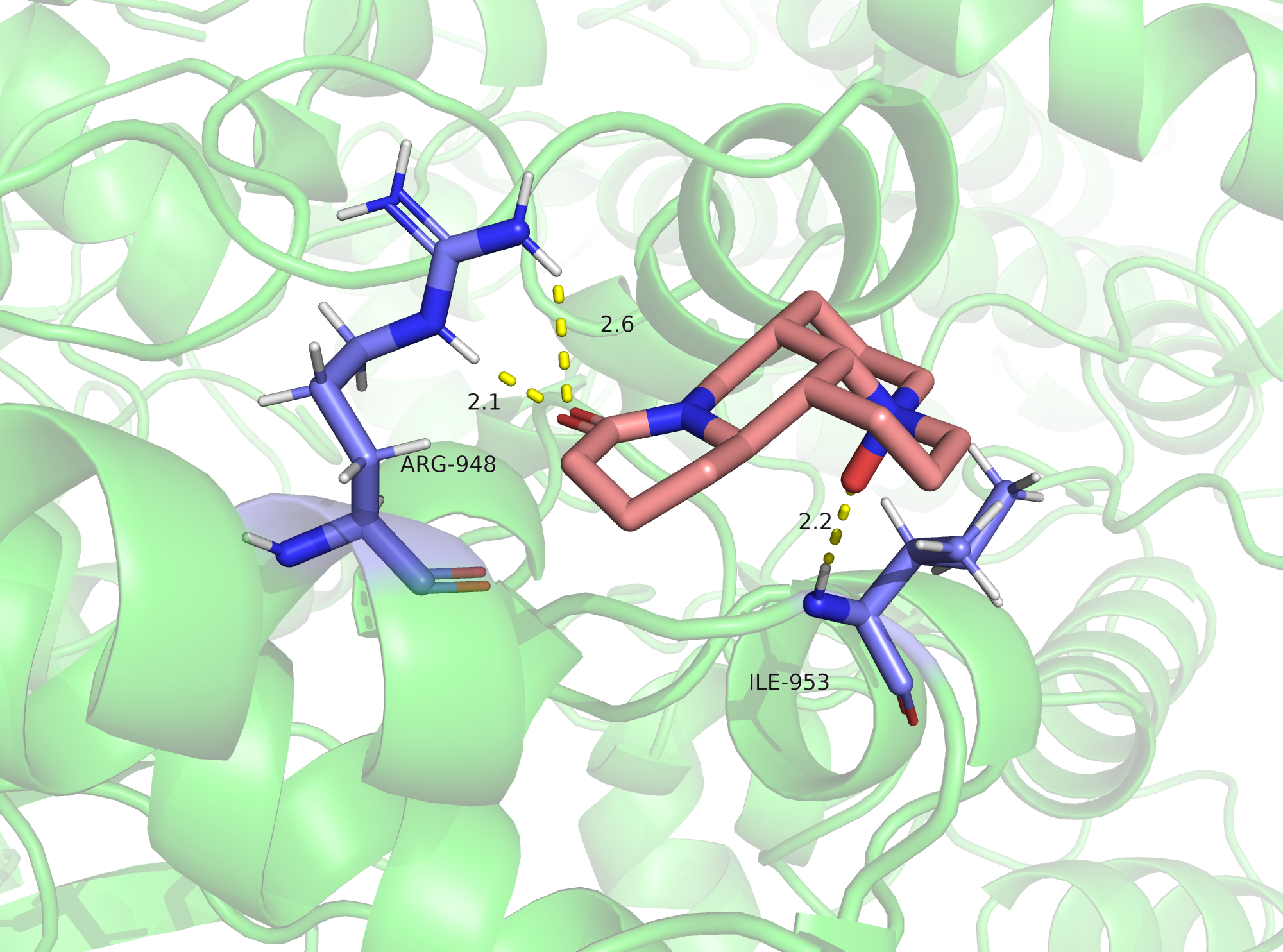

Supplement: Supplementary file 1 [file genes-15-01278-s001.zip › S3/Docking/Isomatrine-EGFR CID_12442899_5I6Z/CID_12442899_5i6z(ray 2000).png]

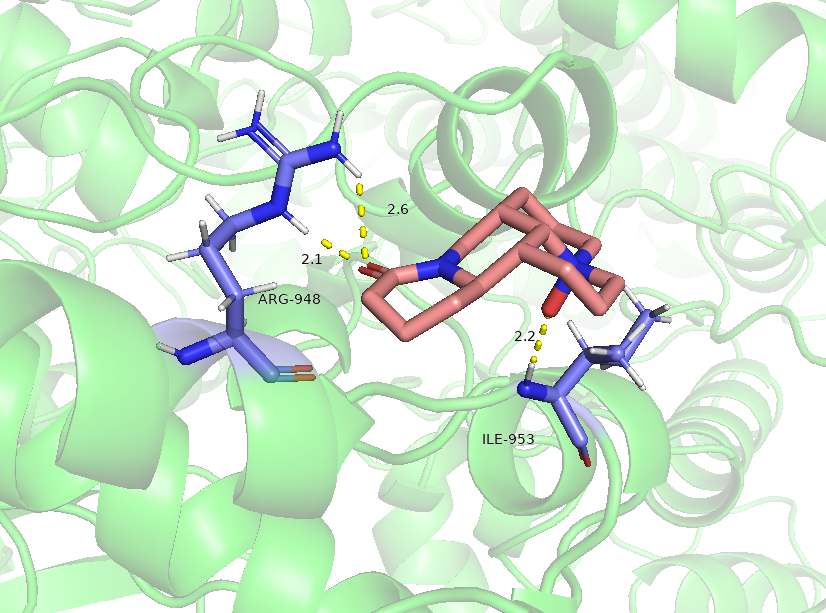

Supplement: Supplementary file 1 [file genes-15-01278-s001.zip › S3/Docking/Isomatrine-EGFR CID_12442899_5I6Z/CID_12442899_5i6z.png]

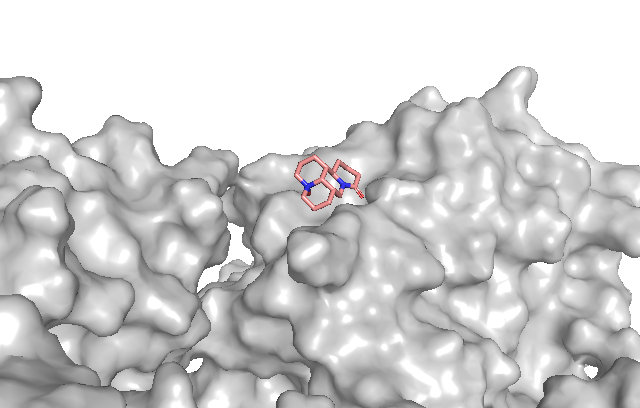

Supplement: Supplementary file 1 [file genes-15-01278-s001.zip › S3/Docking/Isomatrine-EGFR CID_12442899_5I6Z/CID_12442899_5i6z_surface.png]

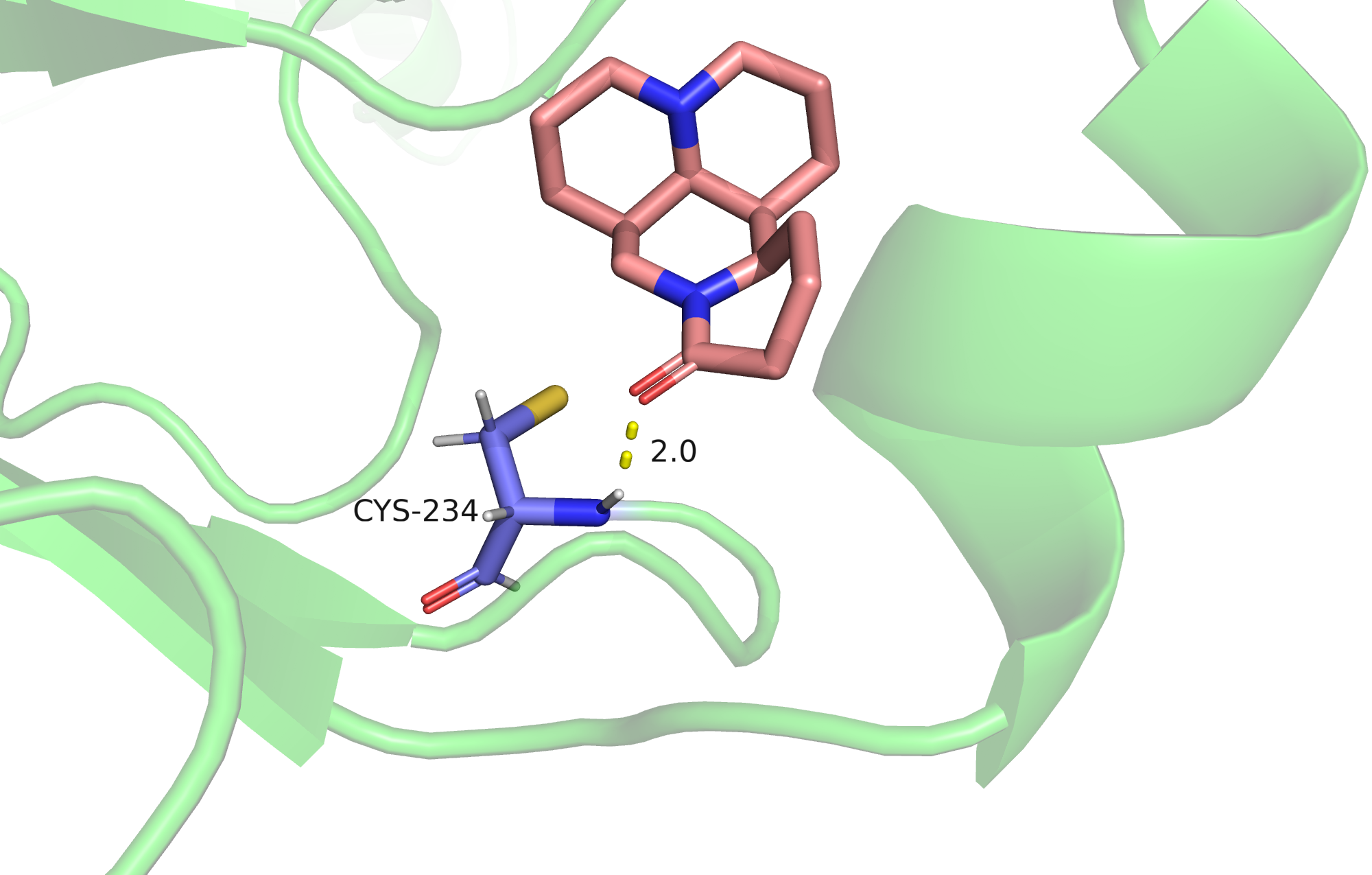

Supplement: Supplementary file 1 [file genes-15-01278-s001.zip › S3/Docking/Isomatrine-ESR1 CID_12442899-3DT3/CID_12442899-3dt3(ray 2000).png]

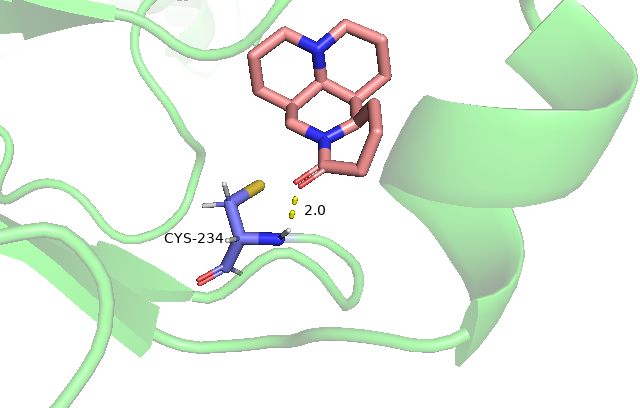

Supplement: Supplementary file 1 [file genes-15-01278-s001.zip › S3/Docking/Isomatrine-ESR1 CID_12442899-3DT3/CID_12442899-3dt3.png]

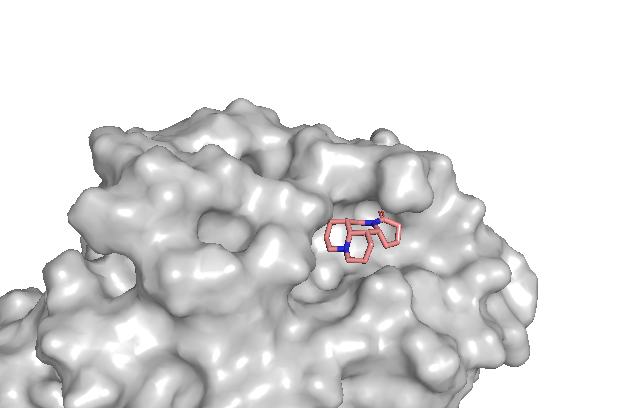

Supplement: Supplementary file 1 [file genes-15-01278-s001.zip › S3/Docking/Isomatrine-ESR1 CID_12442899-3DT3/CID_12442899-3dt3_surface.png]

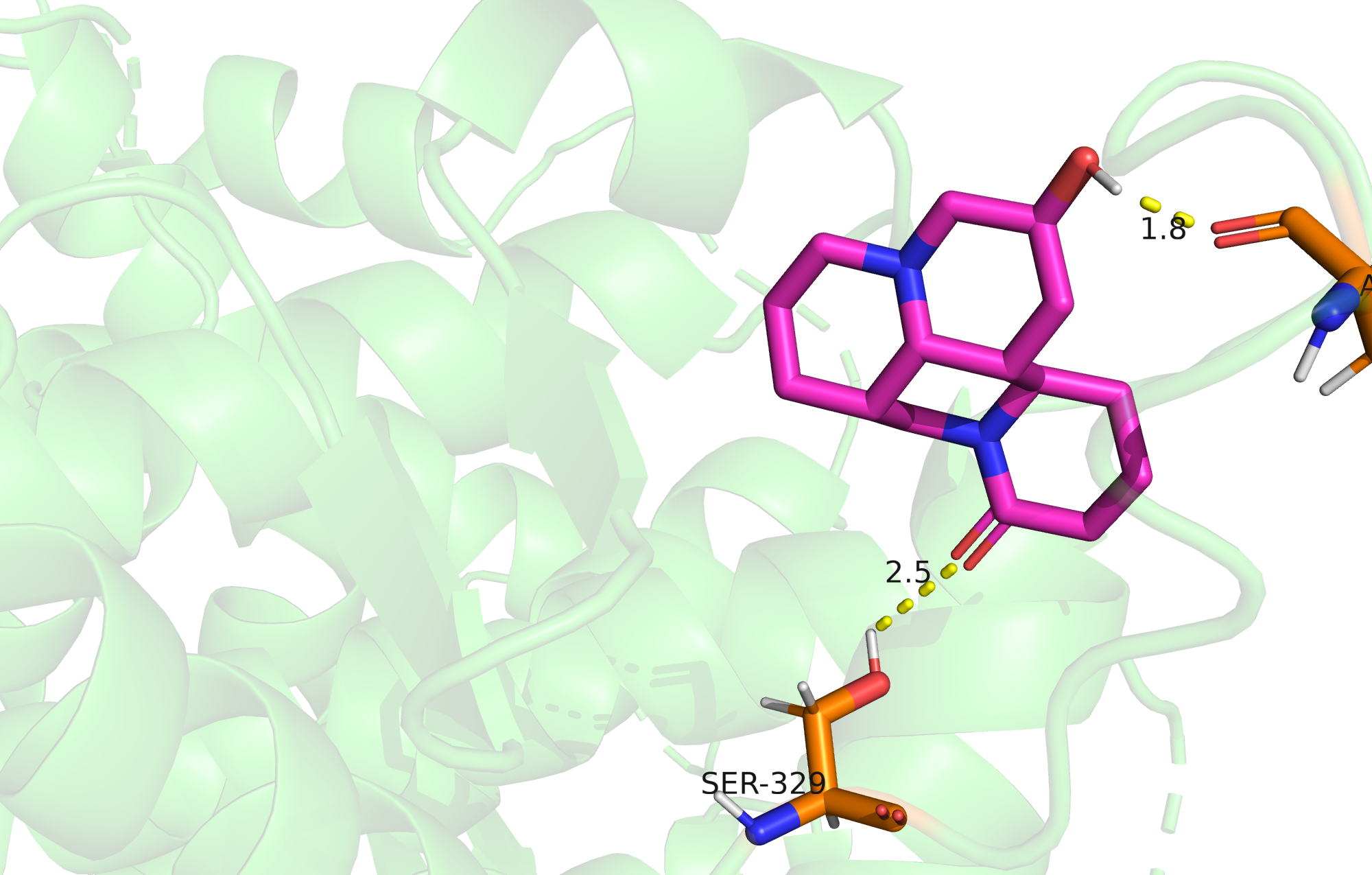

Supplement: Supplementary file 1 [file genes-15-01278-s001.zip › S3/Docking/Matrine-EGFR CID_114850-5I6Z/114850-5i6z(ray 2000).png]

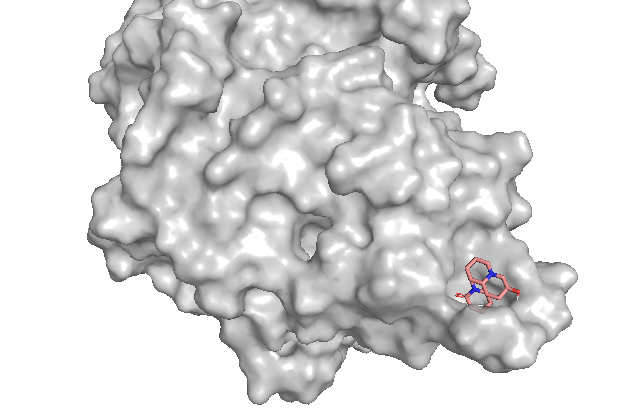

Supplement: Supplementary file 1 [file genes-15-01278-s001.zip › S3/Docking/Matrine-EGFR CID_114850-5I6Z/114850-5i6z-surface.png]

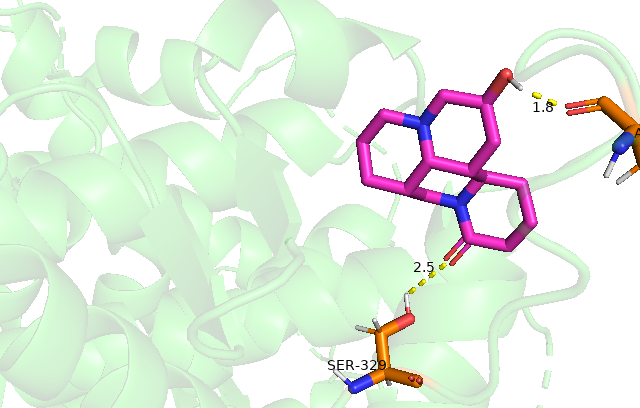

Supplement: Supplementary file 1 [file genes-15-01278-s001.zip › S3/Docking/Matrine-EGFR CID_114850-5I6Z/114850-5i6z.png]

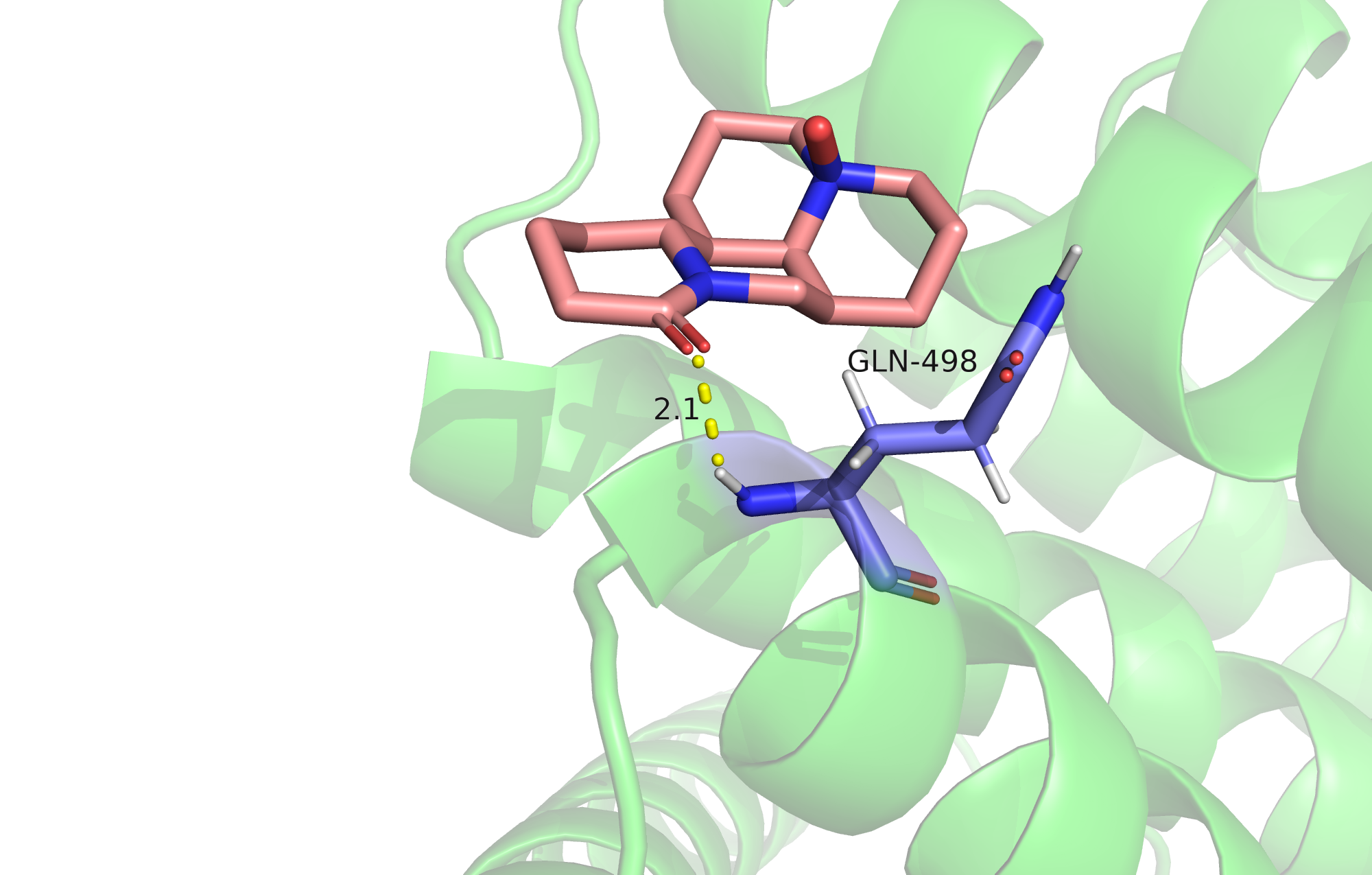

Supplement: Supplementary file 1 [file genes-15-01278-s001.zip › S3/Docking/Matrine-ESR1 CID_114850_3DT3/CID_114850_3dt3(ray 2000).png]

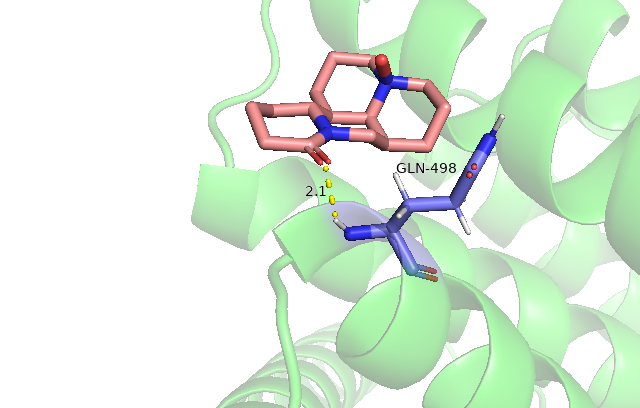

Supplement: Supplementary file 1 [file genes-15-01278-s001.zip › S3/Docking/Matrine-ESR1 CID_114850_3DT3/CID_114850_3dt3.png]

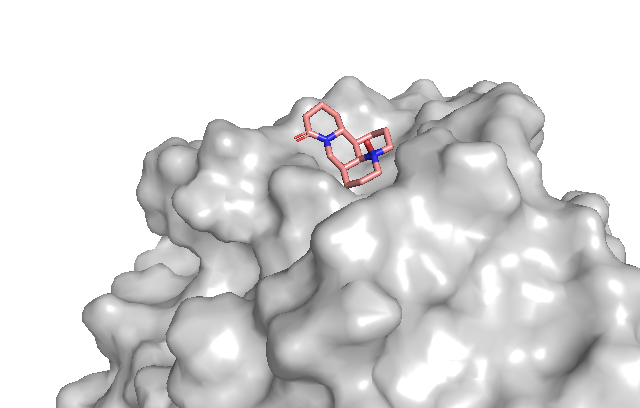

Supplement: Supplementary file 1 [file genes-15-01278-s001.zip › S3/Docking/Matrine-ESR1 CID_114850_3DT3/CID_114850_3dt3_surface.png]

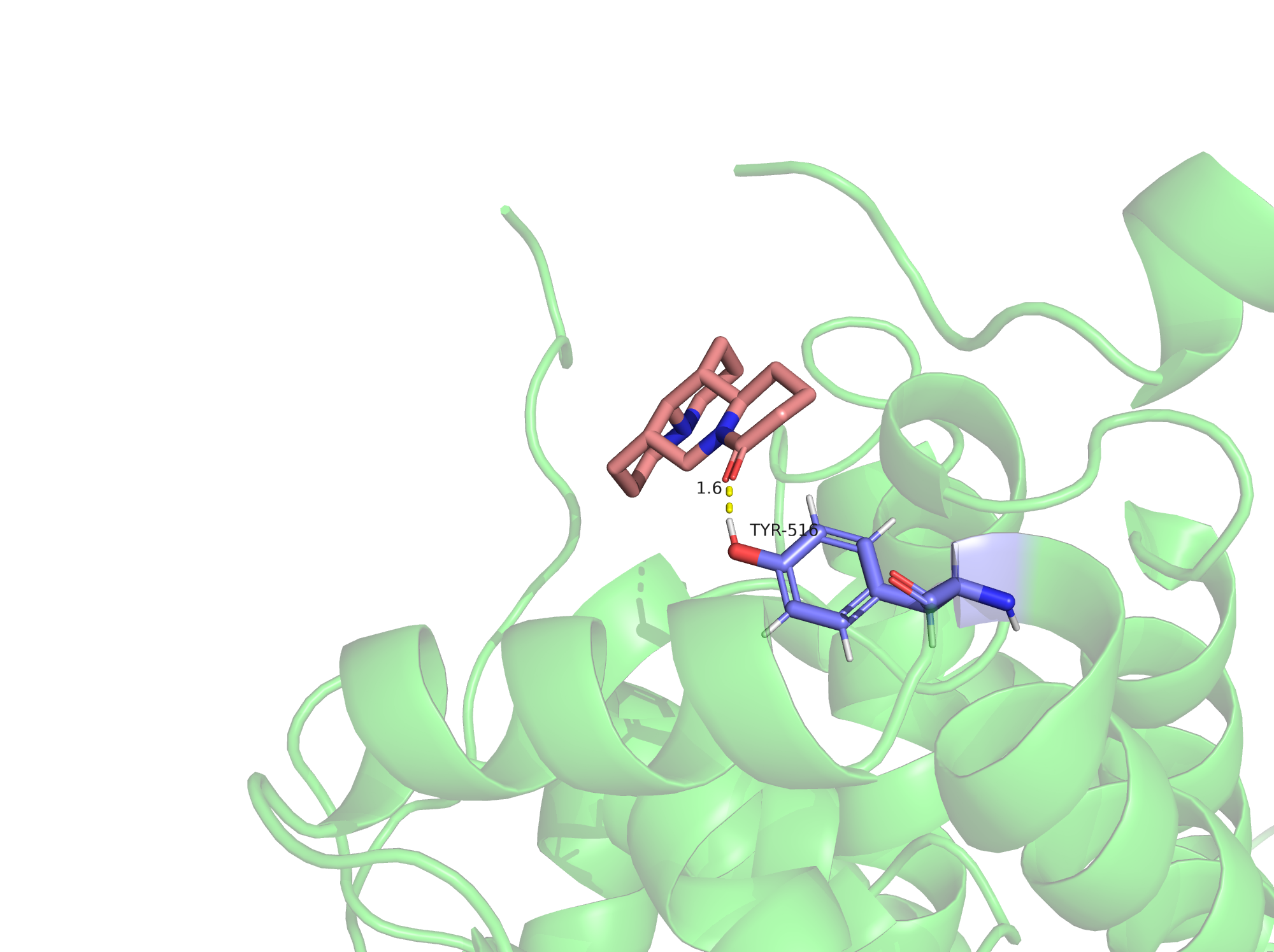

Supplement: Supplementary file 1 [file genes-15-01278-s001.zip › S3/Docking/Oxymatrin-EGFR CID91466_5I6Z/CID91466_5i6z(ray 2000).png]

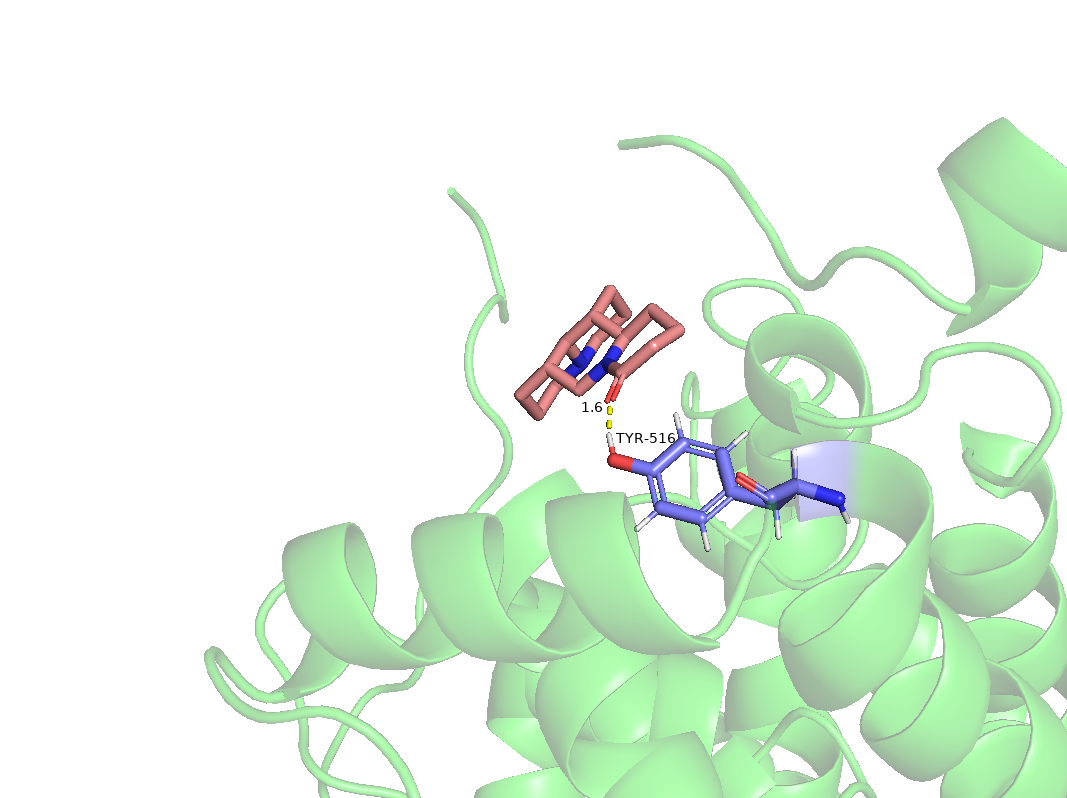

Supplement: Supplementary file 1 [file genes-15-01278-s001.zip › S3/Docking/Oxymatrin-EGFR CID91466_5I6Z/CID91466_5i6z.png]

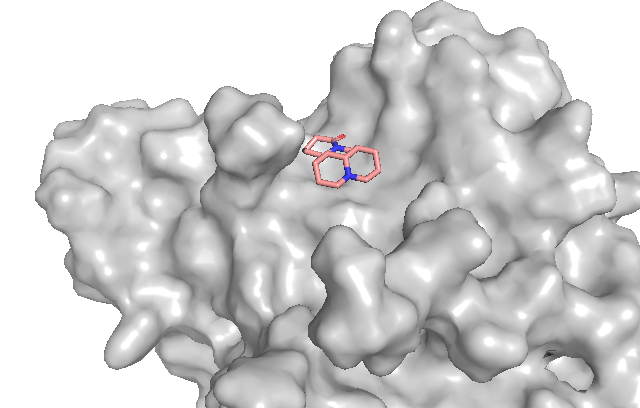

Supplement: Supplementary file 1 [file genes-15-01278-s001.zip › S3/Docking/Oxymatrin-EGFR CID91466_5I6Z/CID91466_5i6z_surface.png]

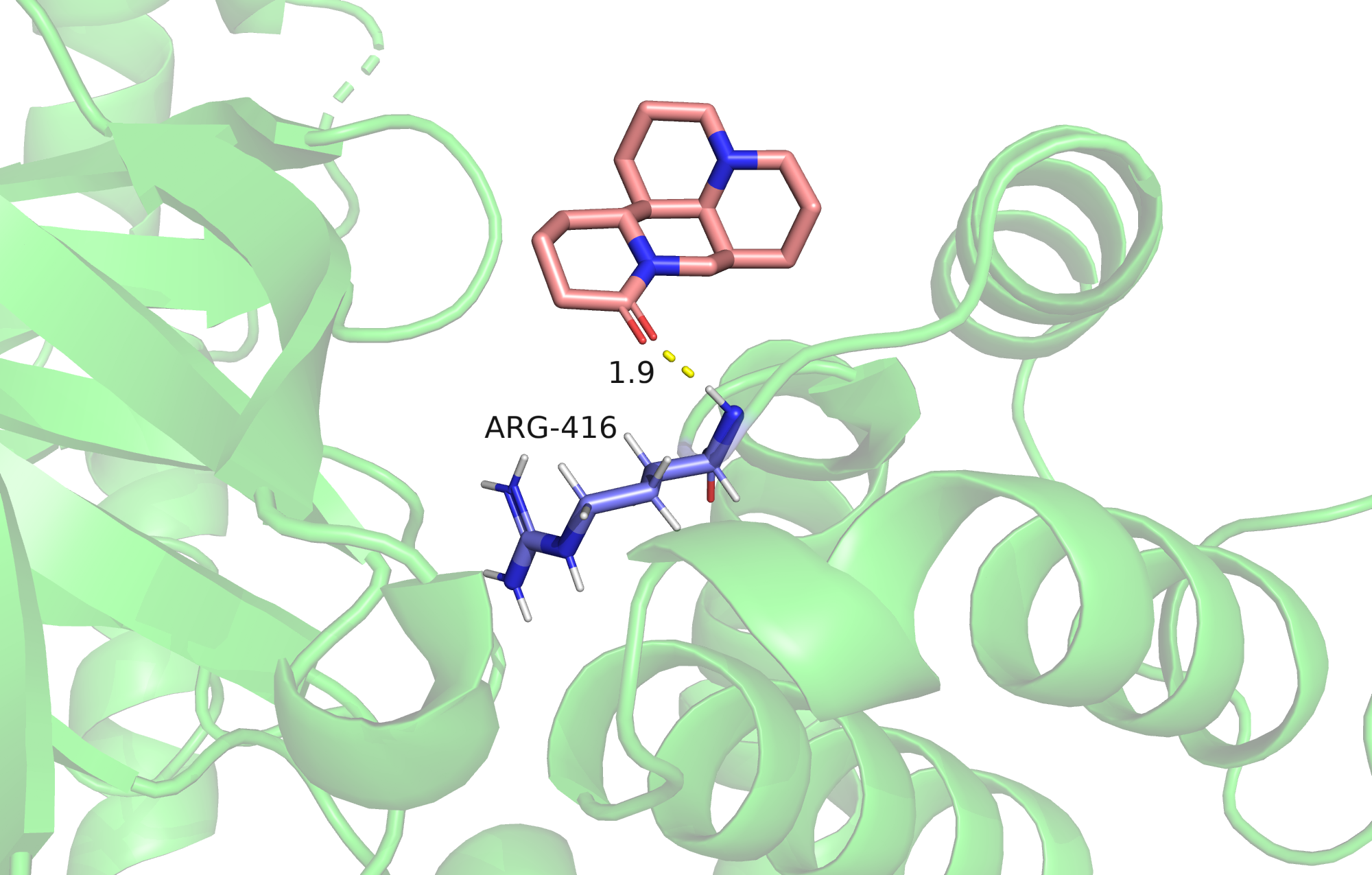

Supplement: Supplementary file 1 [file genes-15-01278-s001.zip › S3/Docking/Oxymatrin-ESR1 CID91466_3DT3/CID91466_3dt3(ray2000).png]

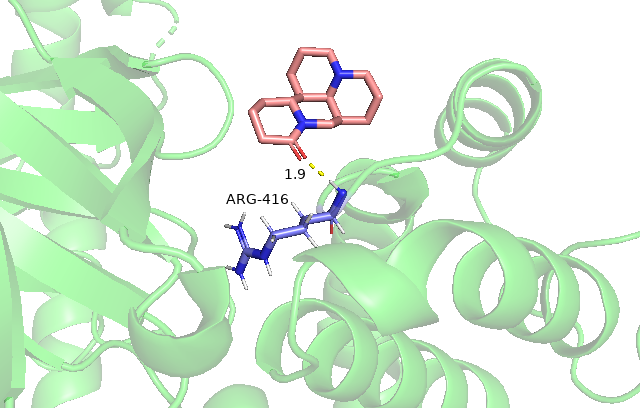

Supplement: Supplementary file 1 [file genes-15-01278-s001.zip › S3/Docking/Oxymatrin-ESR1 CID91466_3DT3/CID91466_3dt3.png]

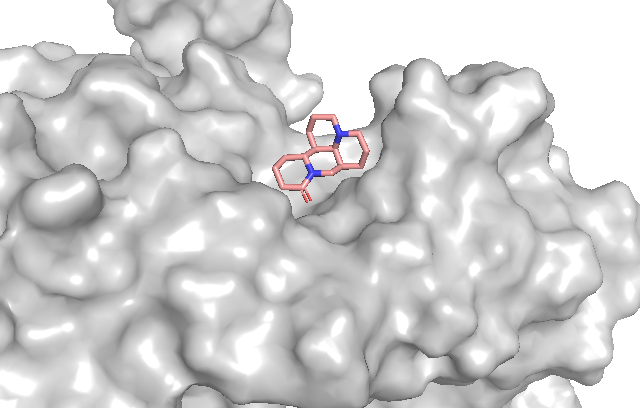

Supplement: Supplementary file 1 [file genes-15-01278-s001.zip › S3/Docking/Oxymatrin-ESR1 CID91466_3DT3/CID91466_3dt3_surface.png]
